# Cascade Reactions Catalyzed by Gold Hybrid Nanoparticles Generate CO Gas Against Periodontitis in Diabetes

**DOI:** 10.1002/advs.202308587

**Published:** 2024-04-22

**Authors:** Yi Wang, Tengda Chu, Ting Jin, Shengming Xu, Cheng Zheng, Jianmin Huang, Sisi Li, Lixia Wu, Jianliang Shen, Xiaojun Cai, Hui Deng

**Affiliations:** ^1^ School and Hospital of Stomatology Wenzhou Medical University Wenzhou Zhejiang 325024 P. R. China; ^2^ Wenzhou Institute University of Chinese Academy of Sciences State Key Laboratory of Ophthalmology Optometry and Vision Science School of Ophthalmology & Optometry School of Biomedical Engineering Wenzhou Medical University Wenzhou Zhejiang 325024 P. R. China

**Keywords:** anti‐inflammatory, cascade reactions, CO gas therapy, hybrid nanoparticle, periodontitis

## Abstract

The treatment of diabetic periodontitis poses a significant challenge due to the presence of local inflammation characterized by excessive glucose concentration, bacterial infection, and high oxidative stress. Herein, mesoporous silica nanoparticles (MSN) are embellished with gold nanoparticles (Au NPs) and loaded with manganese carbonyl to prepare a carbon monoxide (CO) enhanced multienzyme cooperative hybrid nanoplatform (MSN‐Au@CO). The Glucose‐like oxidase activity of Au NPs catalyzes the oxidation of glucose to hydrogen peroxide (H_2_O_2_) and gluconic acid，and then converts H_2_O_2_ to hydroxyl radicals (•OH) by peroxidase‐like activity to destroy bacteria. Moreover, CO production in response to H_2_O_2_, together with Au NPs exhibited a synergistic anti‐inflammatory effect in macrophages challenged by lipopolysaccharides. The underlying mechanism can be the induction of nuclear factor erythroid 2‐related factor 2 to reduce reactive oxygen species, and inhibition of nuclear factor kappa‐B signaling to diminish inflammatory response. Importantly, the antibacterial and anti‐inflammation effects of MSN‐Au@CO are validated in diabetic rats with ligature‐induced periodontitis by showing decreased periodontal bone loss with good biocompatibility. To summarize, MSN‐Au@CO is fabricate to utilize glucose‐activated cascade reaction to eliminate bacteria, and synergize with gas therapy to regulate the immune microenvironment, offering a potential direction for the treatment of diabetic periodontitis.

## Graphical abstract

MSN‐Au@CO nanozymes are designed with enzyme‐like cascade reactions and resultant carbon monoxide (CO) gas release ability. The MSN‐Au@CO nanozymes consume glucose to generate hydroxyl radicals (•OH) that kill bacteria, and the resultant CO can synergistically enhance MSN‐Au to inhibit inflammation through Nrf2 and NF‐κB signaling. The anti‐bacterial and anti‐inflammation effects of MSN‐Au@CO show excellent efficacy in the treatment of periodontitis in diabetic rats.

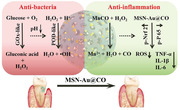

## 1 Introduction

Periodontitis is a chronic inflammatory disorder characterized by the breakdown of tooth‐supporting tissues, loss of gingival attachment to the tooth, and eventually tooth loss. Severe periodontitis is estimated to affect 10.8% of the world's population, ranking as the 6th prevalent disease.^[^ ^1^ ^]^ It is well acknowledged that periodontitis is initiated by the dysbiotic microbiome, but it is the host response that largely determines the extent and severity of alveolar bone loss.^[^ ^2^ ^]^

Diabetes mellitus (DM) is a metabolic disorder syndrome characterized by hyperglycemia. A 2‐way relationship between periodontitis and DM has been established, i.e., hyperglycemia represents a significant risk factor for both the development and progression of periodontitis, and periodontitis contributes to worsening glycemic control and increased risks of diabetic complications.^[^ ^3^ ^]^ Indeed, our previous studies have suggested a potential causal association of fasting glucose with periodontitis,^[^ ^4^ ^]^ and diabetic patients with periodontitis have a higher risk of developing myocardial dysfunction.^[^ ^5^ ^]^ Mechanistically, hyperglycemia and the resultant advanced glycation end‐product formation could enhance bacteria growth and biofilm formation, induce reactive oxygen species (ROS) accumulation, and exacerbate inflammation, tipping the host‐microbial response toward rapid and severe destruction of periodontal tissue.^[^ ^6^ ^]^ This particular microenvironment of diabetic periodontitis characterized by increased glucose accumulation, bacterial growth, and dysregulated immune‐inflammatory response, creates great challenges for conventional periodontal treatment (i.e., mechanical debridement of biofilm and adjunctive antibiotics) to achieve satisfactory results.^[^ ^7^ ^]^ Thus, developing strategies targeting removing bacterial plaque and host modulation that restores the balance between pro‐inflammatory and anti‐inflammatory mediators is critical for the regeneration of diabetic periodontal tissue.

A multi‐enzyme catalytic cascade is a process in which multiple enzymes work together in a specific sequence to catalyze a series of chemical reactions, playing a pivotal role in biological signal transduction and metabolic pathways.^[^ ^8^ ^]^ Unfortunately, natural enzymes are susceptible to various external stressors such as temperature, pH fluctuations, and most organic solvents.^[^ ^9^ ^]^ Recently, there has been significant interest in nanozymes because of their catalytic activity, high stability, and cost‐effectiveness. Some nanozymes such as metal oxides and metal nanomaterials, present multiple enzyme‐like activities under different conditions to address complex pathological microenvironments.^[^ ^10^ ^]^ Among these, gold nanoparticles (Au NPs) have attracted much attention due to their excellent biocompatibility. Notably, Au NPs possess multiple enzyme‐like abilities that allow them to mimic the action of natural glucose oxidase (GOx) and peroxidase (POD) enzymes. This enables Au NPs to convert glucose into both gluconic acid and hydrogen peroxide (H_2_O_2_). Subsequently, the H_2_O_2_ produced can be converted into hydroxyl radicals (•OH), facilitating the consumption of glucose and the death of bacteria.^[^ ^11^ ^]^ Moreover, Au NPs show anti‐inflammatory properties through their ability to inhibit the nuclear factor kappa‐B (NF‐κB) signaling pathway,^[^ ^12^ ^]^ and antioxidant activity by reducing ROS.^[^ ^13^ ^]^ Nevertheless, Au NPs have certain limitations: 1) Uniformly small‐sized Au NPs are required to optimize their enzyme‐like activity; 2) Due to the high surface energy, Au NPs tend to aggregate during catalytic reactions, resulting in reduced catalytic efficiency.

Gas therapy such as hydrogen (H_2_), hydrogen sulfide (H_2_S), carbon monoxide (CO), and nitric oxide (NO), has emerged as an innovative treatment approach due to their critical roles as endogenous signaling molecules in maintaining homeostasis of biological systems.^[^ ^14^ ^]^ Specifically, CO has been proven to show substantial therapeutic potential in cryoprotection, bacterial inhibition, and anti‐inflammation.^[^ ^15^ ^]^ However, like other gaseous molecules, the accurate delivery and controlled release of CO into the diseased tissues present a major challenge. If not properly controlled, an excessive dose of CO can impede mitochondrial respiration by competitively binding to cytochrome c oxidase, irreversibly interfering with its interaction with Oxygen. CO‐releasing molecules (CORMs) have been developed to control the release of CO, triggered by mechanisms such as overproduced H_2_O_2_ ligand exchange reactions, enzyme induction, and near‐infrared (NIR) light.^[^ ^15^, ^16^ ^]^

**Scheme 1:**
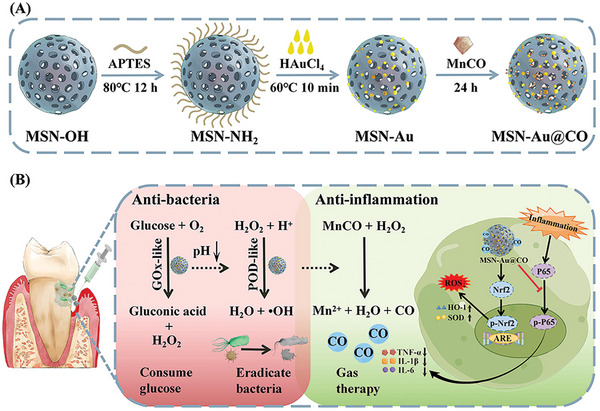
A) Schematic illustration of the construction of MSN‐Au@CO. B) The mechanisms of the cascade reaction present antibacterial and anti‐inflammation effects in the treatment of diabetic periodontitis. Under the nanozyme cascade reaction, MSN‐Au@CO consumes glucose to release •OH that eradicates periodontal bacteria. In addition, the resultant CO enhances MSN‐Au to reduce inflammation by inhibiting P65 nuclear translocation and increasing the antioxidant ability by promoting Nrf2 nuclear translocation.

Herein, ultra‐small Au NPs were coated on mesoporous silica nanoparticles (MSN) as multi‐nanozymes to trigger CO release from manganese carbonyl (MnCO) for the treatment of ligature‐induced‐periodontitis in diabetic rats (**Scheme** **1**). MSN are regarded as promising carriers owing to their excellent biocompatibility, large surface area, adjustable pore diameter, and narrow particle size distribution.^[^ ^17^ ^]^ Au NPs were coated on MSN (MSN‐Au) to improve their stability and enzyme activity. Moreover, MSN‐Au was loaded with MnCO, a CORMs triggered by H_2_O_2_ and •OH (Scheme 1A). In diabetic rats with ligature‐induced periodontitis, Au NPs could oxidize glucose to form gluconic acid and H_2_O_2_ with GOx‐like activity, and subsequently transform H_2_O_2_ into •OH via the POD‐like activity. The cascade reaction and the resultant H_2_O_2_ and •OH not only consumed the accumulated glucose but also eradicated bacteria at the infected sites of diabetic periodontitis.^[^ ^18^ ^]^ Notably, H_2_O_2_ and •OH can render the degradation of MnCO for the in‐situ release of CO gas. The controlled CO release and Au NPs were expected to confer synergistic effects on the inhibition of periodontal inflammation (Scheme 1B). Taken together, our MSN‐Au@CO nanozymes presented with excellent antibacterial and anti‐inflammation activities can be adopted as a new therapeutic nanomaterial to facilitate periodontal healing in diabetic rats.

## 2 Results and Discussion

### 2.1 Synthesis and Characterization of MSN‐Au@CO

**Figure 1:**
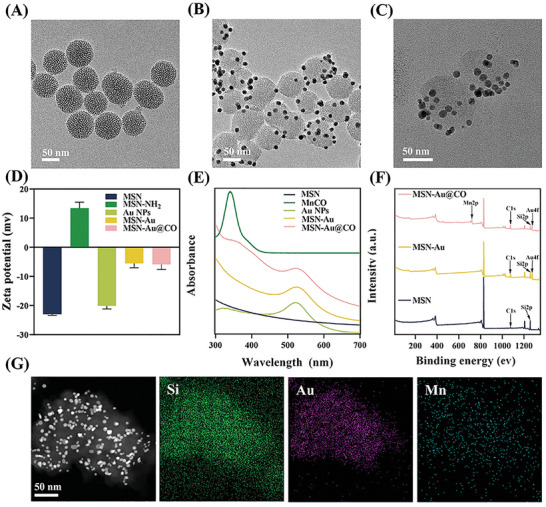
Physicochemical characterization of MSN‐Au@CO. TEM images of A) MSN, B) MSN‐Au, and C) MSN‐Au@CO. D) Zeta potentials of MSN, MSN‐NH_2_, Au, MSN‐Au, MSN‐Au@CO. E) UV–Vis‐NIR absorption spectra of MSN, MnCO, Au, MSN‐Au, MSN‐Au@CO. F) Full survey spectrum of MNS, MSN‐Au, and MSN‐Au@CO. G) EDX mapping of MSN‐Au@CO.

The procedures of MSN‐Au@CO synthesis are illustrated in Scheme 1A. Transmission Electron Microscope (TEM) images showed that the prepared MSN exhibited similarly uniform‐sized spherical nanostructures with radial mesoporous channels. The MSN had an average diameter of 176 ± 9 nm as measured by Dynamic light scattering (DLS) (**Figure** **1**A; Figure S1, Supporting Information). Moreover, the Au NPs coated on the surface of MSN were evenly distributed (Figure 1B). No significant differences in size and morphology were observed between MSN‐Au (average size 182 ± 13 nm) and MSN‐Au@CO (average size 183 ± 14 nm), suggesting that the MnCO loading did not change the original structure of MSN‐Au (Figure 1C; Figure S1, Supporting Information). According to Lambert Beer's law, it was calculated that the drug loading content of Au NPs in MSN‐Au reached 0.05 nmol mg^−1^ (Table S1, Supporting Information). The Zeta potentials of MSN, MSN‐NH_2_, and Au NPs, were −22.9, 13.4, and −20.1 mv, respectively. The Zeta potential of MSN‐Au dropped to −5.5 mv, indicating the successful coating of Au NPs on MSN (Figure 1D). Accordingly, the alterations in the UV/Vis absorption spectra of the MSN, Au NPs, MSN‐Au, MSN‐Au@CO, and MnCO also demonstrated the successful loading of Au NPs and MnCO (Figure 1E). The X‐ray Photoelectron Spectroscopy (XPS) measurements and energy dispersive spectrometer (EDS) mapping were performed to evaluate the elemental composition of MSN, MSN‐Au, and MSN‐Au@CO. The XPS spectra showed the peaks of C 1s, and Si 2p in MSN, MSN‐Au, and MSN‐Au@CO (Figure 1F). The characteristic peak of 84.4 eV from Au 4f was detected in both MSN‐Au and MSN‐Au@CO spectra, while the characteristic peak of 641.5 eV from Mn 2p was observed in MSN‐Au@CO spectra (Figure S2, Supporting Information). Furthermore, the EDS mapping images of MSN‐Au@CO revealed the presence of Si, Au, and Mn. The distribution of Si indicated the nanoparticles were MSN. The Au distribution suggested Au NPs were distributed on the surface of MSN, while the Mn distribution showed MnCO were loaded onto MSN (Figure 1G). Collectively, these characterizations from TEM, DLS, Zeta‐potentials, UV/Vis absorption, XPS spectrum, and EDS mapping confirmed that MSN‐Au@CO were successfully constructed. In order to determine the optimal feed ratio, Inductively Coupled Plasma Optical Emission Spectrometer (ICP‐OES) was employed to detect the levels of Mn elements in MSN‐Au@CO synthesized under various feed ratios. When the feed ratio of MnCO to MSN‐Au was 1:1, the drug loading content of MnCO was measured at 6.27%, and the encapsulation efficiency for MnCO was found to be 6.84% (Figure S3, Supporting Information).

### 2.2 Catalytic Performance and CO Release Behavior of MSN‐Au@CO

**Figure 2:**
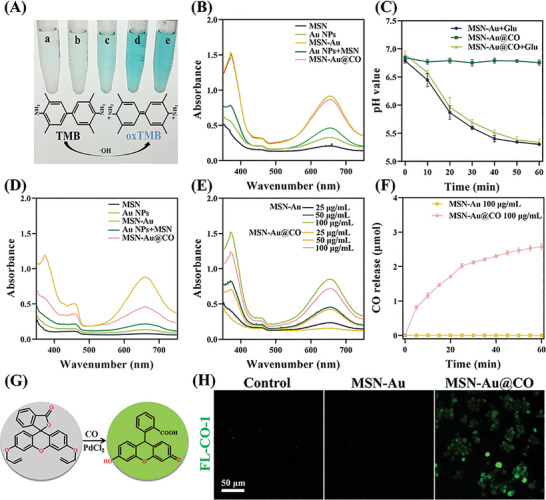
The nanozyme cascade catalytic ability of MSN‐Au@CO. A) Schematic diagram of TMB color rendering principle. a, MSN; b, Au; c, MSN+Au; d, MSN‐Au@CO; e, MSN‐Au. B) The GOx‐like activity of MSN‐Au@CO. C) pH Changes of solutions containing MSN‐Au and MSN‐Au@CO. D) The POD‐like activity of MSN‐Au@CO. (E) The GOx‐like activity of nanomaterials with increasing concentrations. F) Carbon monoxide release profile. G) The schematic diagram of the FL‐CO‐1fluorescence probe. H) Fluorescence imaging of carbon monoxide in macrophages (RAW264.7).

Multi‐nanozymes with GOx‐like and POD‐like catalytic performance were built through the integration of Au NPs on MSN. Au NPs catalyzed glucose into both H_2_O_2_ and gluconic acid (GOx‐like reaction). Subsequently, the resultant H_2_O_2_ was converted into •OH (POD‐like reaction) that exerted antibacterial ability. To assess the GOx‐like activities of MSN‐Au@CO, a 2‐step analytical approach was adopted. The nanoparticles were added into the reaction solution containing glucose, and the produced H_2_O_2_ was identified by observing the oxidation of 3,3′,5,5′‐Tetramethylbenzidine (TMB) (oxTMB with blue color) catalyzed by horse radish peroxidase (HRP). As shown in **Figure** **2**A,B, the solutions containing MSN‐Au and MSN‐Au@CO appeared in blue and exhibited a clear absorption peak at 652 nm, which is the characteristic peak of oxTMB.^[^ ^19^ ^]^ These results confirmed the production of H_2_O_2_ in the first step of the cascade reaction and suggested that MSN‐Au and MSN‐Au@CO were equipped with intrinsic GOx‐like activity. It was found that the pH of the solutions containing MSN‐Au@CO and glucose decreased time‐dependently, while that of the solutions containing MSN‐Au@CO alone was not significantly altered (Figure 2C). The reduction in pH could be attributed to the production of gluconic acid by glycolysis, which was consistent with previous reports.^[^ ^20^ ^]^ To further evaluate the GOx‐like activity quantitively, the Michaelis‐Menten equation (Table S2, Supporting Information) was used to calculate important enzyme kinetic parameters such as Michaelis‐Menten kinetics (*K*_m_) and maximum initial velocity (*V*_max_).^[^ ^21^ ^]^ The value of the *K*_m_ was quantified as 7.44 mM, and that of *V*_max_ was calculated as 0.01 mM min^−1^ (Figure S4A, Supporting Information). Similarly, the POD‐like activity was evaluated by mixing different nanomaterials with H_2_O_2_ using TMB as substrates. As indicated by the absorbance peak at 652 nm in Figure 2D, both MSN‐Au and MSN‐Au@CO catalyzed the oxidation of TMB in the presence of H_2_O_2_, suggesting the POD‐like activity. It should be noted that the MSN‐Au and MSN‐Au@CO group had a higher peak at 652 nm than the Au NPs group, suggesting better catalytic functions. It was possible that after coating on the surface of MSN, the Au NPs were not easily aggregated during catalytic reactions, thereby enhancing the catalytic performance. In addition, the MSN‐Au@CO group had a lower absorbance peak at 652 nm as compared with the MSN‐Au group, suggesting a reduced •OH production, which could be explained by the partial consumption by MnCO. It should be emphasized that the *K*_m_ of MSN‐Au@CO (POD‐like) was 47.29 µM, with a *V*_max_ of 1.01 µM min^−1^ (Figure S4B, Supporting Information). Moreover, we found a positive association of GOx‐ and POD‐like activity with increasing concentrations (25, 50, and 100 µg mL^−1^) of MSN‐Au and MSN‐Au@CO. MSN‐Au/MSN‐Au@CO at a concentration of 100 µg mL^−1^ showed the best catalytic performance (Figure 2E; Figure S5, Supporting Information).

To determine the level of •OH from the cascade reaction, TMB substrate was used to measure the absorbance changes of the solutions with and without MSN‐Au@CO. As shown in Figure S6 (Supporting Information), the absorbance of the solution containing MSN‐Au@CO was time‐dependently increased, indicating the production of •OH.

After confirming the anticipated superior GOx‐ and POD‐like catalytic performance in MSN‐Au@CO, we further assessed the release profile of CO following MnCO oxidation by H_2_O_2_ and •OH.^[^ ^22^ ^]^ The CO generation behavior was analyzed using a CO detector placed in a sealable cylindrical flask containing different nanomaterials in solutions with glucose as the substrate. As illustrated in Figure 2F, MSN‐Au produced almost no CO, while MSN‐Au@CO slowly and stably generated CO for up to 60 min. The CO release profile in response to H_2_O_2_ of our nanozymes with MnCO was validated in Figure S7 (Supporting Information). The amount of CO generated at each time point was calculated as previously described (Table S3, Supporting Information).^[^ ^23^ ^]^ Moreover, the generated CO was identified by the CO fluorescence probe (FL‐CO‐1). This compound enables the detection of CO fluorescence in the presence of PdCl_2_, demonstrating remarkable sensitivity and selectivity (Figure 2G).^[^ ^24^ ^]^ Following the combination of the CO fluorescence probe with the MSN‐Au@CO solution, the fluorescence intensity of the solution increased time‐dependently, indicating the continuous generation of CO (Figure S8, Supporting Information). As shown in Figure 2H, evident green fluorescence was detected in RAW246.7 treated with MSN‐Au@CO, indicating the intracellular presence of CO. In summary, MSN‐Au@CO presented with good GOx‐ and POD‐like activity to catalyze glucose into H_2_O_2_ and •OH, which subsequently oxidize MnCO to release CO slowly and stably.

### 2.3 In Vitro Antibacterial Performance and Biocompatibility of MSN‐Au@CO

**Figure 3:**
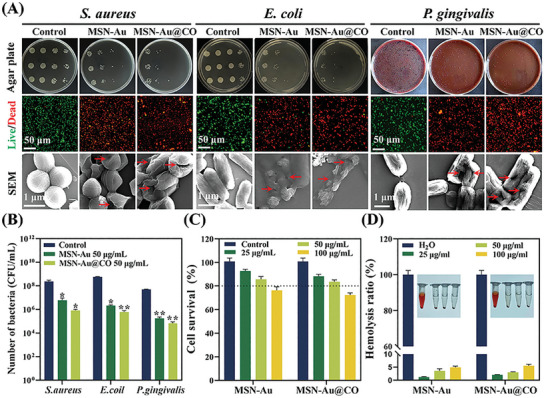
Antibacterial activities of MSN‐Au@CO in vitro. A) Antibacterial activity of different nanoparticles against *S. aureus*, *E. coli*, and *P. gingivalis* as determined by representative images of the plate, bacterial live/dead staining, and SEM analysis. B) Colony quantification diagram of *S. aureus*, *E. coli*, and *P. gingivalis* after different treatments (^*^ Compared with the Control group). Data are presented as mean ± SD (*n* = 3). All *p* values were determined by ANOVA, ^*^ *p* < 0.05, ^**^ *p* < 0.01, and ^***^ *p* < 0.001. C) Determination of cell viability of human periodontal ligament cells (hPDLCs) by MTT method. D) Hemolysis ratio and corresponding photograph.

We have demonstrated the GOx‐ and POD‐like catalytic performance of MSN‐Au@CO, which could be used to convert glucose into H_2_O_2_ and •OH, facilitating bacteria eradication in periodontitis. To confirm this hypothesis, the antibacterial activity of MSN‐Au and MSN‐Au@CO against *Staphylococcus aureus* (*S. aureus*)*, Escherichia coli* (*E. coli*), and *Porphyromonas gingivalis* (*P. gingivalis*) was evaluated. As shown in Figure S9A (Supporting Information), in the presence of glucose, both MSN‐Au and MSN‐Au@CO presented comparable dose‐dependent antibacterial activity against *S. aureus, E. coli*, and *P. gingivalis*. It was found that 50 µg mL^−1^ of MSN‐Au and MSN‐Au@CO conferred outstanding bactericidal effects with >99.9% reduction for *S. aureus* and *E. coli* and 99% reduction for *P. gingivalis* (**Figure** **3**A,B). In contrast, we did not observe any significant antibacterial effect of MSN‐Au and MSN‐Au@CO in the absence of glucose (Figure S9B, Supporting Information). The antibacterial performance was further confirmed by Live/Dead staining results in Figure 3A. Almost all *S. aureus*, *E. coli*, and *P. gingivalis* treated by MSN‐Au and MSN‐Au@CO in the presence of glucose were stained with propodium iodide (PI), emitting a strong red fluorescence. To further explore the mechanisms of antibacterial activity, scanning electron microscope (SEM) was performed to observe the morphology of the bacteria after the indicated treatment. As shown in Figure 3A, the cell membranes of *S. aureus*, *E. coli*, and *P. gingivalis* were smooth and intact in the Control group. MSN‐Au and MSN‐Au@CO conferred apparent damage on *S. aureus*, *E. coli*, and *P. gingivalis*, as evidenced by the shrinkage and rupture of bacterial cell membranes. Following the rupture of cell membranes, the bacterial protein could be released into the supernatant. We, therefore, quantified the protein level in the supernatant to assess the protein leakage. As shown in Figure S10 (Supporting Information), MSN‐Au induced protein leakage in *S. aureus* (0.12 mg mL^−1^), *E. coli* (0.10 mg mL^−1^), and *P. gingivalis* (0.14 mg mL^−1^), MSN‐Au@CO led to a similar amount of protein leakage. Together, the above results demonstrated that both MSN‐Au and MSN‐Au@CO (50 and 100 µg mL^−1^) had excellent antibacterial properties. While previous studies have shown the antibacterial effects of CO, no significant synergistic effects were observed in our study.^[^ ^25^ ^]^ It could be explained by the steady and slow CO production profile of MSN‐Au@CO so that the concentration of CO did not reach the level required to exert antibacterial effects.^[^ ^26^ ^]^

Biocompatibility is essential for the successful application of biomedical materials. Human Periodontal ligament cells (hPDLCs), which constitute the majority of the periodontal ligament that maintains the integrity and function of the periodontium,^[^ ^28^ ^]^ were employed to test the biocompatibility of the synthesized nanoparticles (Figure 3C). Following 24 h of co‐culture with 50 µg mL^−1^ of MSN‐Au@CO, the cell viability of hPDLCs was slightly decreased to 83.5% ± 1.9%, which could be attributed to the H_2_O_2_ from GOx‐like activity. Notably, the cell survival rate was increased to 88.5% ± 4.1% when the co‐culture time was extended to 48 h, indicating good biocompatibility of MSN‐Au@CO at the concentration of 50 µg/mL (Figure S11A, Supporting Information). Similar results were found in the Live/Dead staining assay. 50 µg/mL of MSN‐Au and MSN‐Au@CO hardly affected the normal morphology of hPDLCs, and only a few red fluorescences were observed. While the concentrations of MSN‐Au and MSN‐Au@CO were increased to 100 µg/mL, the treated cells began to show moderate morphological changes and higher intensity of red fluorescence, as compared with the Control group (Figure S11B, Supporting Information). As shown in Figure 3D, MSN‐Au, and MSN‐Au@CO did not produce obvious hemolysis in red blood cells with a concentration of up to 100 µg mL^−1^. According to the aforementioned results, 50 µg mL^−1^ of MSN‐Au/MSN‐Au@CO showed good antibacterial activity and high biocompatibility, this concentration was therefore chosen for the following experiments.

### 2.4 In Vitro Anti‐Inflammatory Performance

**Figure 4:**
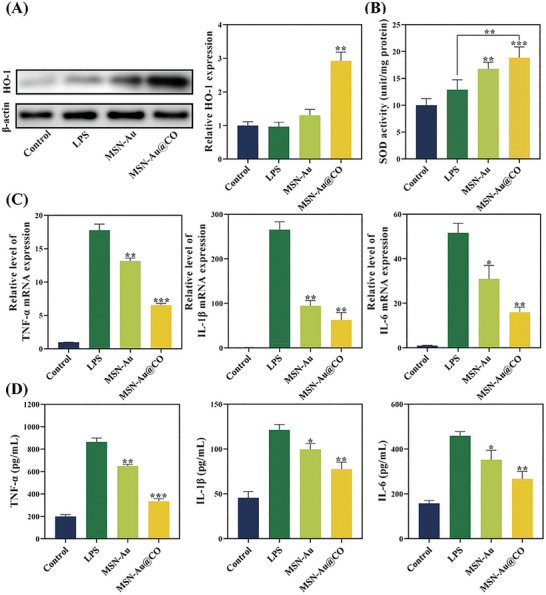
Antioxidant and anti‐inflammatory effects of MSN‐Au@CO in macrophages challenged with LPS. A) Western blot analysis of HO‐1 in macrophage with different treatment (^*^ Compared with the control group). B) SOD enzyme activity of macrophages with different treatment (^*^ Compared with the control group). C) Relative mRNA levels of pro‐inflammatory genes TNF‐α, IL‐1β, and IL‐6 were determined by q‐PCR (^*^ Compared with the LPS group). D) Concentrations of TNF‐α, IL‐1β, and IL‐6 in the cell supernatant measured by ELISA (^*^ Compared with the LPS group). Data are presented as mean ± SD (*n* = 3). All *p* values were determined by ANOVA, ^*^ *p* < 0.05, ^**^ *p* < 0.01, and ^***^ *p *< 0.001.

Successful management of periodontitis requires not only the elimination of periodontal plaque but also host modulation by inhibition of oxidative stress and inflammation.^[^ ^28^, ^29^ ^]^ Previous studies have demonstrated the significant anti‐inflammatory ability of Au NPs by regulating inflammatory cytokines and macrophage polarization.^[^ ^12^ ^]^ Moreover, the administration of CO at low concentrations has been adopted to reduce inflammation and ROS in various tissues.^[^ ^30^ ^]^ Therefore, our synthesized MSN‐Au@CO was expected to weaken inflammation and oxidative stress in inflamed periodontal tissues. RAW264.7, which releases a series of inflammatory mediators under the stimulation of lipopolysaccharide (LPS), was adopted for in vitro anti‐inflammation experiments.^[^ ^31^ ^]^ To verify our conjecture, we examined the anti‐inflammatory and anti‐oxidant effects of MSN‐Au@CO in RAW 246.7 macrophages challenged by LPS. It was found that both MSN‐Au and MSN‐Au@CO upregulated the protein expression of Heme oxygenase‐1 (HO‐1) and Superoxide dismutases (SOD) activity, which are known to counteract oxidative stress (**Figure** **4**A,B). Interestingly, we did not observe significant effects of LPS on HO‐1 protein expression and the intracellular SOD activity. Several previous studies have reported different results on the HO‐1 and SOD expression in LPS‐challenged macrophages.^[^ ^32^ ^]^ The discrepancy may arise from the experimental settings such as the origin, concentration, and induction time of the LPS used. To show the changes in intracellular ROS levels, 2′,7′‐Dichlorodihydrofluorescein diacetate (DCFH‐DA) probes were used to detect intracellular ROS levels after different treatments. It was shown that the LPS‐induced ROS level was significantly inhibited by the treatment of MSN‐Au@CO (Figure S12, Supporting Information). The antioxidant activity was more pronounced in MSN‐Au@CO than MSN‐Au, suggesting the possible synergistic anti‐oxidant effect from Au NPs and CO. The mRNA levels of TNF‐α, IL‐1β, and IL‐6 were significantly decreased by both MSN‐Au and MSN‐Au@CO, as depicted in Figure 4C. Moreover, ELISA results further confirmed the reduction of inflammatory cytokines in RAW 246.7 treated by MSN‐Au and MSN‐Au@CO (Figure 4D). Notably, compared with MSN‐Au, MSN‐Au@CO induced a greater reduction of TNF‐α, IL‐1β, and IL‐6 at both transcript and protein levels, suggesting the synergistic anti‐inflammatory effect from Au NPs and CO.

**Figure 5:**
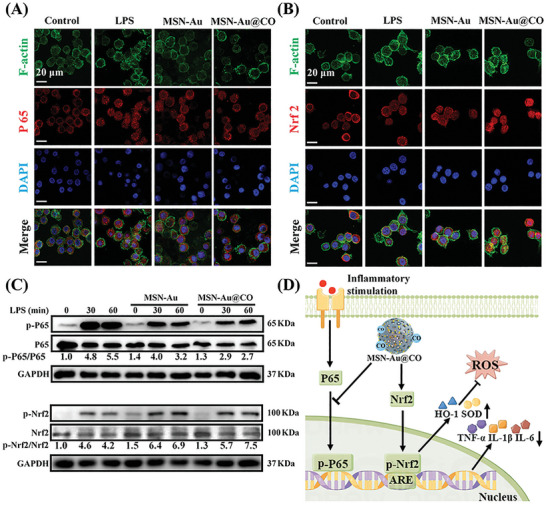
The effects of MSN‐Au@CO on NF‐κB and Nrf2 signaling in macrophages challenged by LPS. A) Immunofluorescence images of P65 (red) and (B) Nrf2 (red) in macrophages of different treatment groups. Nuclei were stained with DAPI (blue), and myofilament protein was stained with F‐actin (green). C) Western blot analysis in macrophages of different treatment groups. D) The potential antioxidant and anti‐inflammatory mechanism of MSN‐Au@CO.

NF‐κB is a transcription factor that regulates the expression of genes that play a crucial role in inflammation. Upon inflammatory stimulation, P65 (major subunit of NF‐κB) is phosphorylated and translocated into the nucleus, allowing it to exert its transcriptional control over target genes (e.g., TNF‐α, IL‐1β, and IL‐6) involved in immune and inflammatory responses.^[^ ^33^ ^]^ Nuclear factor erythroid 2‐related factor2 (Nrf2) is a key regulatory protein involved in the cellular defense against oxidative stress. When cells sense oxidative stress, Nrf2 can be phosphorylated and subsequently translocated into the cell nucleus, where it activates the transcription of a wide array of genes encoding antioxidant enzymes.^[^ ^34^ ^]^ To confirm our hypothesis that NF‐κB and Nrf2 signaling were involved in the anti‐inflammatory and anti‐oxidant effects of MSN‐Au and MSN‐Au@CO, the intracellular localization of P65 and Nrf2 was evaluated by immunofluorescence. P65 was translocated into nuclei upon LPS stimulation. After treatment with MSN‐Au and MSN‐Au@CO, P65 was relocated into the cytoplasm, indicating the inhibition of NF‐κB signaling (**Figure** **5**A). In addition, MSN‐Au and MSN‐Au@CO induced Nrf2 nuclear translocation, as evidenced by the enhanced Nrf2 staining (red) overlapped with DAPI (blue) (Figure 5B). Next, a western blot was performed to determine the phosphorylation of P65 and Nrf2 (Figure 5C). It was found that MSN‐Au@CO induced a more pronounced inhibition of p‐P65, as compared with MSN‐Au and the Control group. As expected, LPS triggered Nrf2 phosphorylation and its subsequent nuclear translocation, which was in line with previous reports.^[^ ^35^ ^]^ Moreover, MSN‐Au@CO increased the p‐Nrf2/Nrf2 ratio to a greater extent in reference to the MSN‐Au and Control group. The more pronounced Nrf2 phosphorylation could be attributed to the CO production and MSN‐Au that work synergistically to promote antioxidant capability. Collectively, our results showed that the synergistic anti‐inflammatory and anti‐oxidant effects could be mediated by NF‐κB and Nrf2 signaling (Figure 5D).

### 2.5 In Vivo Therapeutic Efficacy for Ligature‐Induced‐Periodontitis

**Figure 6:**
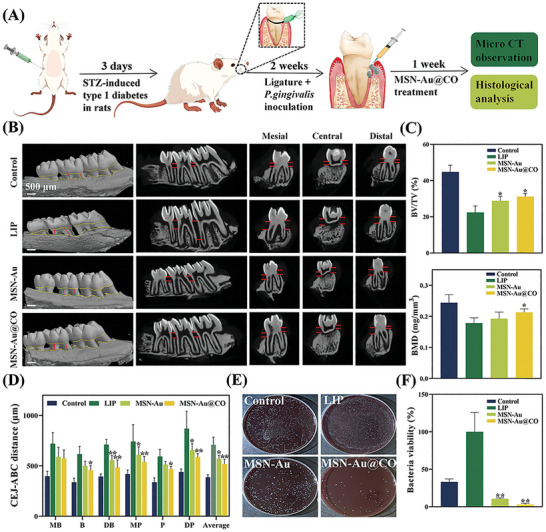
Efficacy of MSN‐Au@CO in the treatment of ligature‐induced periodontitis (LIP) in diabetic rats. A) Schematic diagram of experimental design. B) 3D reconstructed digitized images, mesiodistal section images, and bucco‐palatal section images of M2 analyzed by Micro‐CT. C) Bone‐related parameters (BV/TV) and BMD in different groups were analyzed by Micro‐CT (^*^ Compared with the LIP group). D) CEJ‐ABC distance at six sites of the maxillary second molars in different groups analyzed by Micro‐CT (^*^ Compared with the LIP group). E) Representative images of plate samples after different treatments. F) Bacteria viability from infected tissues upon different treatments was measured by colony counts (^*^ Compared with the LIP group). LIP, ligature‐induced‐periodontitis. Data are presented as mean ± SD (*n* = 3–5). All p values were determined by ANOVA, ^*^ *p* < 0.05, ^**^ *p* < 0.01, and ^***^ *p* < 0.001.

As illustrated in schematic **Figure** **6**A, the in vivo therapeutic efficacy of MSN‐Au and MSN‐Au@CO were evaluated in diabetic rats with ligature‐induced periodontitis (LIP). Figure S13A (Supporting Information) showed a sustained blood glucose level above 16.6 mmol L^−1^ in all rats 3 days after Streptozotocin (STZ) injection. Thereafter, experimental periodontitis was established by placing ligature subgingivally around the maxillary second molars (M2).

To evaluate the periodontal bone loss in diabetic rats, micro‐CT was conducted and the cemento⁃enamel junction‐alveolar bone crest (CEJ‐ABC) distance at 6 sites was measured. The viewing angle and magnification of the micro‐CT image were pre‐adjusted to ensure the accuracy of the measurement. 3D reconstructed images and different cross‐sectional images are shown in Figure 6B. The bone volume per tissue volume (BV/TV, %) and the bone mineral density (BMD, mg mm^−3^) of the M2 mesiodistal alveolar crest were measured to determine the bone quality. As displayed in Figure 6C,D, LIP profoundly decreased BV/TV, BMD, and increased CEJ‐ABC distance, showing substantial bone loss and the establishment of experimental periodontitis. Importantly, MSN‐Au@CO significantly alleviated LIP‐induced bone loss, as indicated by the increased BV/TV and BMD, as well as the reduced CEJ‐ABC distance. As for the MSN‐Au treated rats, significantly increased BV/TV and decreased CEJ‐ABC distance were noted as compared with the LIP group, while the difference in BMD did not reach statistical significance. These micro‐CT findings suggested a positive effect of MSN‐Au and MSN‐Au@CO on delaying bone loss in diabetic rats with experimental periodontitis.

**Figure 7:**
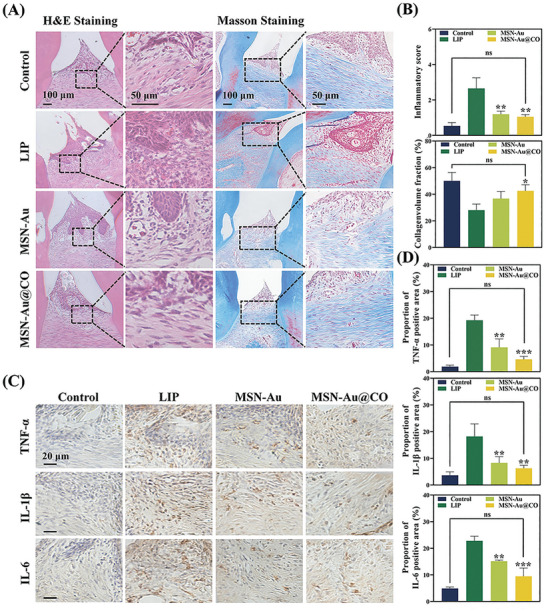
Histological analyses of papillary connective tissue and alveolar bone around the M2 after different treatments. A) Representative images of H&E staining and Masson staining on the periodontium after the treatment. B) Semiquantitative assessment of inflammatory cells and collagen volume fraction (^*^ Compared with the LIP group). C) The immunohistochemical staining of TNF‐α, IL‐β, and IL‐6 in the gingival tissues. D) Semi‐quantitative assessment of TNF‐α, IL‐β, and IL‐6 proportion of positive area (*Compared with the LIP group). LIP, ligature‐induced periodontitis. Data are presented as mean ± SD (*n* = 3). All p values were determined by ANOVA, ns, not significant, ^*^ *p* < 0.05, ^**^ *p* < 0.01, and ^***^ *p* < 0.001.

To assess the antibacterial properties of MSN‐Au and MSN‐Au@CO in vivo, the subgingival plaque was collected and cultured anaerobically. There was a large number of bacterial colonies from the LIP group. As expected, MSN‐Au and MSN‐Au@CO treatment significantly eradicated ≈89.5% and 97.9% of the bacteria, indicating an effective antibacterial ability (Figure 6E,F). Hematoxylin and Eosin staining (H&E) and Masson's staining were performed to evaluate the infiltration of inflammatory cells and the repair of damaged periodontal tissue respectively. Compared with the Control group, we observed a larger number of immune cell infiltration, as well as more disordered and degraded periodontal ligament fibers between M2 and M3 of LIP‐treated rats. In the MSN‐Au/MSN‐Au@CO group, there was little immune cell infiltration and the periodontal ligament fibers were arranged densely and regularly (**Figure** **7**A,B). Consistent with the in vitro anti‐inflammatory effects of MSN‐Au/MSN‐Au@CO, the immunohistochemistry staining (IHC) revealed that MSN‐Au/MSN‐Au@CO significantly downregulated the inflammatory cytokine expressions in the gingival tissues between M2 and maxillary third molars (M3), as compared with LIP group (Figure 7C,D). To sum up, both MSN‐Au and MSN‐Au@CO were demonstrated to be effective in preventing periodontal bone loss possibly through immunoinflammatory regulation and bacterial eradication. Furthermore, MSN‐Au@CO showed a superior in vivo therapeutic efficacy than MSN‐Au, indicating the potential synergistic effect of CO and Au NPs.

### 2.6 In Vivo Biosafety Evaluation

The biocompatibility of MSN‐Au@CO was further evaluated in vivo. During the experiment, there was no significant difference in body weight among the rats in each group (Figure S13B, Supporting Information). In addition, H&E staining was performed on the main organs (i.e., heart, liver, spleen, lung, and kidney) of rats after indicated treatments, and no obvious damage and pathological changes were found following in vivo application of MSN‐Au@CO (Figure S14, Supporting Information).

## 3 Conclusion

In this work, a novel multi‐enzyme synergistic nanoplatform based on CO gas therapy (MSN‐Au@CO) was designed. Benefitting from the Au NPs on the surface of MSN, the obtained MSN‐Au@CO presented efficient multi‐enzyme catalytic activity. Specifically, MSN‐Au@CO catalyzed the oxidation of glucose to generate H_2_O_2_ (GOx‐like) and produced abundant •OH (POD‐like). This multi‐enzyme activity aided in lowering the local glucose levels and effectively eliminating bacteria. It is worth noting that MnCO loading in MSN‐Au@CO enabled the release of CO in situ in response to H_2_O_2_ and •OH. The resultant CO and MSN‐Au showed synergistic anti‐inflammatory effects via Nrf2 and NF‐κB signaling pathways. Utilizing these remarkable properties, the administration of MSN‐Au@CO in diabetic rats with periodontitis significantly eliminated bacteria, alleviated periodontal inflammation and inhibited bone loss with good biocompatibility. To sum up, MSN‐Au@CO could be a multifunctional nanozyme platform as a promising strategy for the treatment of diabetic periodontitis.

## 4 Experimental Section

### Materials

Cetyltrimethylammonium bromide (CTAB), anhydrous ethanol, HAuCl_4_ •3H_2_O, Manganese carbonyl (MnCO), 3‐aminopropyltrimethoxysilane (APTES, >99%), Trisodium citrate, tetraethylorthosilicate (TEOS), Tannic acid, LPS from *E. coli* O111:B4, and ammonia (NH_3_·H_2_O) were purchased from Sigma‐Aldrich Chemical Co. Methylthiazolyldiphenyl‐tetrazolium bromide (MTT), BCA protein assay kit, DCFH‐DA, Triton X‐100, propidium iodide (PI) and calcein acetoxymethyl ester (Calcein‐AM), 4,6‐diamino‐2‐phenylindole (DAPI), were purchased from Beyotime Biotechnology Co. ELISA kits for the measurement of TNF‐α, IL‐6, and IL‐1β were purchased from Neobioscience Co., Ltd. (Shenzhen, China). Fetal bovine serum (FBS), Dulbecco's modified Eagle medium (DMEM), and penicillin‐streptomycin (P&S) were acquired from Thermo Scientific (Beijing, China). *Escherichia coli* (*E. coli*, ATCC 25 923), *Staphylococcus aureus* (*S. aureus*, ATCC 25 922), *Porphyromonas gingivalis* (*P. gingivalis*, W83), and RAW264.7 murine macrophages were obtained from American Type Culture Collection (ATCC). hPDLCs were obtained from Pricella (Cat No. CP‐H234). For all experiments, deionized (DI) water (18.2 MΩ cm^−1^) was employed. Additionally, all other chemicals and reagents utilized were of analytical grade and used without further modification.

### Synthesis of MSN‐NH_2_

APTES‐modified MSN were synthesized following the previously reported procedure.^[^ ^36^ ^]^ Briefly, CTAB (5 mM) was dissolved in 100 mL of NH_3_·H_2_O (0.17 M) solution at 50 °C with stirring until clarification. Then 2 mL of TEOS (0.88 m) ethanol solution was added to the solution with intense stirring. The system was stirred overnight. Next, the synthesized MSN were centrifuged at 14 000 rpm for 10 min and washed alternately with water and anhydrous ethanol 5 times. The acid‐alcohol extraction method was used to remove the CTAB template. The synthesized samples were added to an acidic ethanol solution (1 mL of HCl‐10 mL of ethanol) and refluxed for 2 h under stirring at 60 °C. The MSN was dispersed in methylbenzene and refluxed at 80 °C with APTES for 12 h. Finally, the resultant product was collected through centrifugation, washed 3 times with ethanol, and subsequently dried under a vacuum.

### Synthesis of MSN‐Au and MnCO Loading

MSN‐Au was synthesized by sodium citrate reduction method.^[^ ^37^ ^]^ Solution A was prepared by dispersing MSN‐NH_2_ and chloroauric acid (HAuCl_4_ 0.01 g mL^−1^) was dispersed in DI water and ultrasonicated for 30 min. Trisodium citrate (0.01 g mL^−1^), K_2_CO_3_ (0.14 g mL^−1^), and tannic acid (0.01 g mL^−1^) were added to DI water as reaction solution B. Then solution B was added into solution A with stirring at 700 rpm, and a temperature of 60 °C for 10 min. The mixed solution was centrifuged to collect the sediment. The sediment was washed with DI water 3 times and dried under a vacuum to obtain the MSN‐Au. Then, MSN‐Au was dispersed in the above MnCO methanol solution under intense stirring for 24 h. ICP‐OES was used to quantify the contents of the Mn element in MSN‐Au@CO. The drug loading content 1) and the drug encapsulation efficiency 2) of MnCO in MSN‐Au@CO were calculated according to the following formulas:

(1) $$\;LE_{MnCO}\%\;=\frac{M_{MnCO}}{M_{1}}\;\times 100\%$$

(2) $$\;EE_{MnCO}\%\;=\frac{M_{MnCO}}{M_{0}}\;\times 100\%$$

Note: *LE_MnCO_*% is the drug loading content of the MnCO in MSN‐Au@CO, *M_MnCO_* is the quality of MnCO in MSN‐Au@CO, and *M_1_* is the sampling quality of MSN‐Au@CO. *EE_MnCO_*% is the drug encapsulation efficiency of the MnCO in MSN‐Au@CO, and *M_0_* is the mass of MnCO initially put into the reaction.

### Characterization

The hydrodynamic particle size and zeta potential of nanoparticles were determined on a Malvern Zetasizer Nano series (Zetasizernano ZS, UK). UV–Vis spectra were recorded by using a UV‐3600 UV‐VIS‐NIR (Japan). The elemental chemical states were used by X‐ray photoelectron spectroscopy (XPS, EscaLab 250Xi, China). The morphology of nanoparticles was characterized by high‐resolution transmission electron microscopy (TEM, FEI Tecnai G2 F20, USA), and the corresponding energy dispersivespectrometer (EDS) mapping was used for elemental distribution analysis.

### Gox‐Like Activity

The Gox‐like activities of MSN, Au NPs, MSN‐Au, and MSN‐Au@CO were assessed through a 2‐step approach. In the first step, the nanozymes were coincubated with glucose, leading to a reaction (glucose + O_2_ → H_2_O_2_ + gluconic acid). Subsequently, TMB and HRP were added, and the resulting supernatant was analyzed using a UV–Vis spectrophotometer. The kinetic parameters of the GOx‐like were determined using the Michaelis‐Menten equation (Table S2, Supporting Information). The pH of the solution was determined by a pH meter (AZ8601, AZ Instrument Corp, China). To further detect the production of •OH during the enzyme cascade reaction of MSN‐Au@CO, the sodium acetate buffer (pH 5.0) with MSN‐Au@CO, glucose, and TMB, and monitored the absorbance changes in the solution was supplemented.

### POD‐Like Activity

In the presence of H_2_O_2_, POD‐like activity facilitates the oxidation of TMB, leading to the formation of OxTMB and the subsequent manifestation of a blue color. MSN, Au NPs, MSN‐Au, MSN‐Au@CO, TMB, and H_2_O_2_ were mixed in acetate buffer (pH 4.0). After 30 min, the supernatant was detected by UV‐vis spectrophotometer. The Michaelis‐Menten equation (Table S2, Supporting Information) was employed to calculate the kinetic parameters and maximum velocity of the POD‐like activity of MSN‐Au@CO.

### CO Generation Behavior

In order to evaluate the CO release ability of MSN‐Au@CO in vitro. Different concentrations of MSN‐Au@CO were added to the PBS containing 20 mmol L^−1^ glucose and placed in a sealed glass container together with the carbon monoxide gas detector. Carbon monoxide concentration (PPM) was recorded at each time point. The amount of CO released by MSN‐Au@CO was calculated using the previously reported formula, and the corresponding formulas are shown in Table S3 (Supporting Information).^[^ ^23^ ^]^

Similarly, after mixing MSN‐Au@CO with the FL‐CO‐1, the fluorescence intensity at each time point using an ultraviolet fluorescence spectrophotometer was detected.

The intracellular CO release was assessed using a typical CO fluorescence probe.^[^ ^24a^ ^]^ RAW264.7 (2 × 10^5^ cells well^−1^) was initially seeded in 48‐well plates and cultured for 24 h at 37 °C with 5% CO_2_. Then, the culture medium was replaced with a fresh medium containing either MSN‐Au or MSN‐Au@CO. After coculture for 4 h, the medium was removed and a fresh medium containing FL‐CO‐1 (1 µg mL^−1^ in DMSO) was added. Following 3 washes with PBS, the intracellular fluorescence was examined using an inverted fluorescence microscope.

### In Vitro Antibacterial Activity

The *S. aureus* and *E. coli* serve as the representative strains of Gram‐positive and Gram‐negative bacteria, respectively. *P. gingivalis* promotes the development of periodontitis, serving as the most prevalent etiological agent of periodontal disease.^[^ ^38^ ^]^ Thus, these 3 bacteria were used to evaluate the in vitro antibacterial ability of MSN‐Au@CO by bacterial colony counting. First, the 3 types of bacteria were cultured overnight at 37 °C in a tryptone soy broth (TSB) medium. The bacterial suspension was centrifuged and resuspended in PBS to obtain a concentration of 10^8^ (CFU mL^−1^). Then the bacterial suspension was treated by MSN‐Au/MSN‐Au@CO with or without glucose (20 mM) at 37 °C for 2 h, using different nanozymes concentrations. Next, the bacterial suspension was subjected to stepwise serial dilution ranging from 10^6^ to 10^3^, and cultured on TSB plates at 37 °C overnight. Finally, the resulting colonies were counted, with the remaining bacterial quantity being expressed as log_10_x. To perform bacterial live‐dead staining, the bacterial suspension was centrifuged and underwent 2 washes with PBS after various treatments. Then, the bacteria suspension was cocultured with the SYTO 9/PI Bacterial Double Stain Kit (MKBio, China) in the dark at 37 °C for 1 h. Following this, the bacteria were washed 3 times with PBS and examined using an inverted fluorescence microscope (ZEISS, Germany). For SEM (Hitachi, Japan) images of bacteria, the bacterial samples were fixed with 4% paraformaldehyde for 12 h after different treatments, and then dehydrated in a series of ethanol concentrations (20%, 40%, 60%, 80%, and anhydrous) before being dropped on the silicon to SEM observation.

### Biosafety Assessment

Human periodontal ligament cells (hPDLCs) were cultured in DMEM supplemented with 10% (v/v) FBS and 1% (v/v) P&S, at 37 °C with 5% CO_2_ environment. The MTT assay was used to detect the cytotoxicity of MSN‐Au/MSN‐Au@CO in vitro. The hPDLCs were seeded in a 96‐well plate at a density of 5 × 10^3^ (cells/well) for 24 h. Following PBS washes, the cells were exposed to DMEM containing varying concentrations of nanozymes for 24 h under a 37 °C incubation with a 5% CO2 atmosphere. After coculture, MTT (10 µL) was added to the cells, which were then coincubated for 4 h at 37 °C. The absorbance at 490 nm was measured to determine cell viability. For the Live/Dead cell staining assay, cells were first incubated with the nanozymes for 24 h. Afterward, the cells were stained with Calcein AM/PI and observed using an inverted fluorescence microscope (ZEISS Germany).

To assess the blood compatibility of the nanozymes, a hemolysis test was conducted. Blood was mixed with PBS containing varying concentrations of MSN‐Au/MSN‐Au@CO and incubated at 37 °C for 1 h. Then the solution was centrifuged at 2500 rpm for 5 min and the absorbance at 540 nm was measured. DI water and PBS were used as positive and negative controls, respectively. The hemolysis rate was calculated according to the previously reported formula (Table S4, Supporting Information).^[^ ^39^ ^]^

### In Vitro Anti‐Oxidative Stress Effect

The antioxidant capacity was assessed to measure the levels of intracellular ROS in macrophages with DCFH‐DA probes (Beyotime, China). Briefly, RAW264.7 (2 × 10^5^ cells well^−1^) was seeded in 48‐well plates and incubated with LPS for 24 h. Subsequently, the medium was replaced by fresh DMEM containing MSN‐Au/MSN‐Au@CO for 6 h, followed by DCFH‐DA for 0.5 h in the dark. The fluorescence was observed by an inverted fluorescence microscope (ZEISS Germany).

In order to further evaluate the antioxidative stress ability of MSN‐Au@CO, this study measured the protein expression levels of HO‐1 and the total superoxide dismutase. Western blotting was used to detect the expression of HO‐1. Briefly, cells were coincubated with different materials for 24 h. The total proteins were extracted on ice using a radioimmunoprecipitation assay (RIPA) buffer. Subsequently, the protein samples were subjected to electrophoresis on a 10% sodium dodecyl sulfate‐polyacrylamide gel electrophoresis (SDS‐PAGE) and transferred onto vinylidene fluoride (PVDF) membranes. To block the membranes, a solution of 5% BSA dissolved in PBS was applied at room temperature for 1 h. The PVDF membranes, containing the proteins were then incubated with a primary antibody specifically targeting HO‐1 (1:3000, Cell Signaling Technology) overnight at 4 °C. Following this, the PVDF membranes were incubated with a secondary antibody at room temperature for 1 h. The PVDF membranes were exposed using the BeyoECL Moon kit (Beyotime, China). The total SOD was detected using the Total Superoxide Dismutase Assay Kit with WST‐8 (Beyotime Institute of Biotechnology, China).

### In Vitro Anti‐Inflammatory Effect

The mRNA expression of anti‐inflammatory genes (TNF‐α, IL‐1β, IL‐6) was measured through quantitative Real‐time polymerase chain reaction PCR (q‐PCR) assay. First, RAW 264.7 cells (5 × 10^5^ cells well**^−^** ^1^) were seeded in 6‐well plates and stimulated with 200 ng mL**^−^** ^1^ of LPS for 24 h. Then the cells were treated with DMEM containing MSN‐Au/MSN‐Au@CO (50 µg mL**^−^** ^1^) for 6 h. Finally, the supernatant was collected to measure the levels of TNF‐α, IL‐1β, and IL‐6 using an ELISA assay, while the cells were harvested for mRNA expression analysis via q‐PCR. Normal Macrophage cells, LPS‐stimulated RAW264.7 without treatment, acted as negative and positive controls, respectively. The primer sequences used are shown in Table S5 (Supporting Information).

Western blot analysis was used to detect the related expression levels of p‐P65 and P65, Nrf2, p‐Nrf2, and GAPDH. RAW264.7 macrophage cells were cultured and seeded at 8 × 10^5^ cells per well in a 6‐well plate. The cells were pretreated by medium with MSN‐Au/MSN‐Au@CO (50 µg mL**^−^** ^1^) for 24 h and then stimulated by LPS (200 ng mL**^−^** ^1^) for 0, 30, and 60 min as processing groups. The target protein was detected using the above‐mentioned method of Western blot.

To examine the expression and location of P65 and Nrf2, immunofluorescence staining was performed on macrophage cells as described above. The macrophage cells were fixed in 4% paraformaldehyde for 10 min. Then the cells were incubated with an anti‐P65 antibody (1:200, Cell Signaling Technology) or anti‐Nrf2 antibody (1:200, Cell Signaling Technology) overnight at 4 °C. After washing the cells with PBS, they were incubated with Cy3‐labeled goat anti‐rabbit IgG antibody (1:200, Beyotime Biotechnology) and subsequently visualized under a confocal laser scanning microscope (CLSM).

### In Vivo Therapeutic Efficacy on Diabetic Rats with Periodontitis

All the animal experiments were approved by the Experimental Animal Ethics Review Committee of Wenzhou Medical University and performed in compliance with the Code of Conduct for the Care and Use of Experimental Animals. Before the experiment, the animals were acclimatized for 1 week and randomly divided into 4 groups (*n* = 5) in a blinded manner. After fasting overnight, rats were administered STZ (40 mg kg^−1^, Solarbio, China) intraperitoneally to induce diabetes. Rats with a plasma glucose concentration greater than 16.6 mmol L^−1^ after 3 days were considered diabetic.^[^ ^40^ ^]^ In order to confirm the stability of the diabetic model, the blood glucose of the corresponding rats every 3 days was measured. To induce ligature‐induced periodontitis in diabetic rats, a silk thread infused with *P. gingivalis* (10^8^ CFU mL**^−^** ^1^) was gently ligated around the gingiva region encircling the maxillary second molar (M2) for 2 weeks. After ligature removal, different nanoparticles were locally administered with a micro‐syringe every 2 days 3 times. Two days after the last nanoparticle administration, cotton swaps were used to collect subgingival plaque samples from the maxillary second molar for anaerobic culture. The colonies were counted to assess the in vivo antibacterial ability of the nanoparticles.^[^ ^41^ ^]^ All rats were then euthanized for the following experiments.

Alveolar bone resorption was evaluated through micro‐CT analysis. A section of the maxillae including all 3 molars, together with alveolar bone and attached gingival tissue were collected and fixed in 4% paraformaldehyde for 24 h. Subsequently, the specimen was scanned using a micro‐CT scanner (Bruker, Germany). Alveolar bone resorption was assessed by measuring the distance between the CEJ‐ABC, as well as by calculating the bone volume to tissue volume ratio (BV/TV, %) using 3D digitized images.

For histological analysis, the samples were decalcified using 10% ethylene diamine tetra acetic acid (EDTA) for 4 weeks, followed by sequential dehydration using ethanol (30%, 50%, 70%, 85%, 95%, and anhydrous). The samples were then treated with xylene and embedded in paraffin. Thin sections (5 µm) were obtained from the paraffin blocks and stained with hematoxylin & eosin and Masson to assess the histological alterations. Inflammatory cells were first evaluated using a semi‐quantitative scoring method, where 0 represents negative, 1 was less than 30% of the affected area, 2 was 30%–60% inflammatory cells, and 3 represents many inflammatory cells (> 60%).^[^ ^42^ ^]^

The samples also were collected for anti‐inflammatory (TNF‐α, IL‐1β, IL‐6) evaluation by immunohistochemistry staining. In brief, the sections underwent cold antigen retrieval using trypsin followed by an overnight incubation with the primary antibody. The primary antibody was subsequently removed, followed by incubation with a biotinylated goat‐anti‐rabbit secondary antibody at room temperature, which was further developed with a diaminobenzidine (DAB) solution. The final step involved re‐staining the samples using Mayer's hematoxylin.^[^ ^43^ ^]^

### Statistic Analysis

All quantitative data were expressed as mean ± SD from at least triplicate measurements. Student's *t*‐test was adopted to evaluate the differences between the 2 groups. The comparisons between multiple groups were analyzed by one‐way analysis of variance (ANOVA), followed by post‐hoc Turkey's test using Graphpad Prism 8.4.0 (Graphpad software, USA). A 2‐tailed difference with ns means not significant, ^*^ *p* < 0.05, ^**^ *p* < 0.01, and ^***^ *p *< 0.001 were considered statistically significant.

## Conflict of Interest

The authors declared no conflict of interest.

## Author Contributions

Y.W. and T.C. contributed equally to this work and are joint first authors. Y.W. and T.C. conceived the project, designed the experiments, conducted the experiments, analyzed the data, and drafted the manuscript. T.J., S.X., C.Z., J.H., S.L., and L.W. conducted the experiments and analyzed the data. J.S. supervised the study, conceived the project, and revised the manuscript. X.C. and H.D. conceived the project, supervised the study, acquired the funding, and drafted and revised the manuscript.

## Supporting information

Supporting Information

## Data Availability

The data that support the findings of this study are available from the corresponding author upon reasonable request.
