# Supplementary material for: Cascade Reactions Catalyzed by Gold Hybrid Nanoparticles Generate CO Gas Against Periodontitis in Diabetes
Source: Adv Sci (Weinh). 2024 Apr 22;11(24):2308587. doi: 10.1002/advs.202308587 (PMC11199988; doi:10.1002/advs.202308587)
Supplement: Supplementary file 1 — Supporting Information [file ADVS-11-2308587-s001.docx]

**Cascade reactions catalyzed by gold hybrid nanoparticles generate CO gas against periodontitis in diabetes**

Yi Wang, Tengda Chu, Ting Jin, Shengming Xu, Cheng Zheng, Jianmin Huang, Sisi Li, Lixia Wu, Jianliang Shen*, Xiaojun Cai*, Hui Deng*

Yi Wang, Tengda Chu, Ting Jin, Shengming Xu, Cheng Zheng, Jianmin Huang, Sisi Li, Lixia Wu, Xiaojun Cai*, Hui Deng*

School and Hospital of Stomatology, Wenzhou Medical University, Wenzhou, Zhejiang, P. R. China

E-mail: huideng@wmu.edu.cn (Hui Deng); cxj520118@wmu.edu.cn (Xiaojun Cai)

Jianliang Shen*

Wenzhou Institute, University of Chinese Academy of Sciences, State Key Laboratory of Ophthalmology, Optometry and Vision Science, School of Ophthalmology & Optometry, School of Biomedical Engineering, Wenzhou Medical University, Wenzhou, Zhejiang, P. R. China

E-mail: shenjl@wiucas.ac.cn

**Supplemental Figures:**


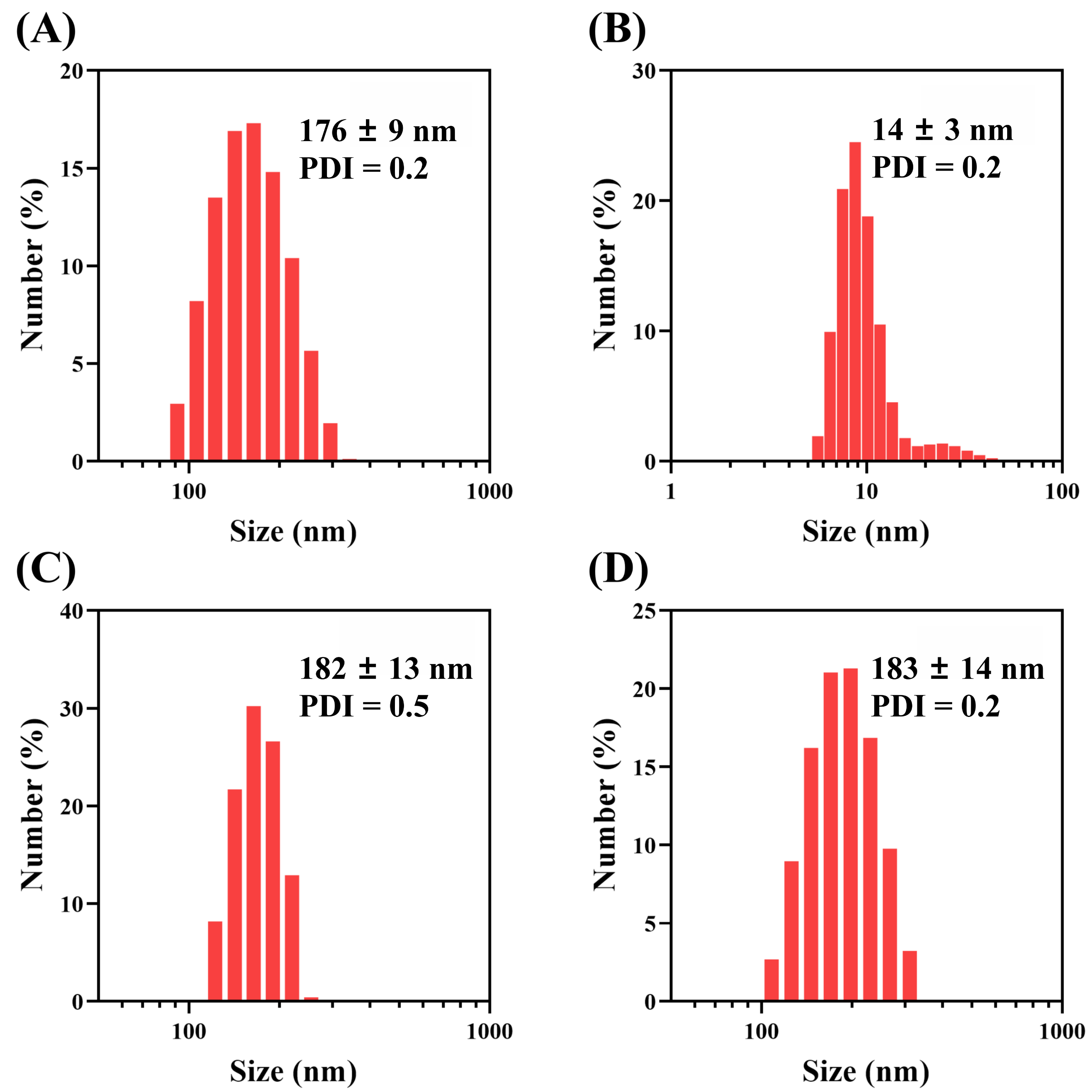


**Figure S1** Hydrodynamic size distribution of (A) MSN, (B) Au NPs, (C) MSN-Au, (D) MSN-Au@CO measured by DLS.


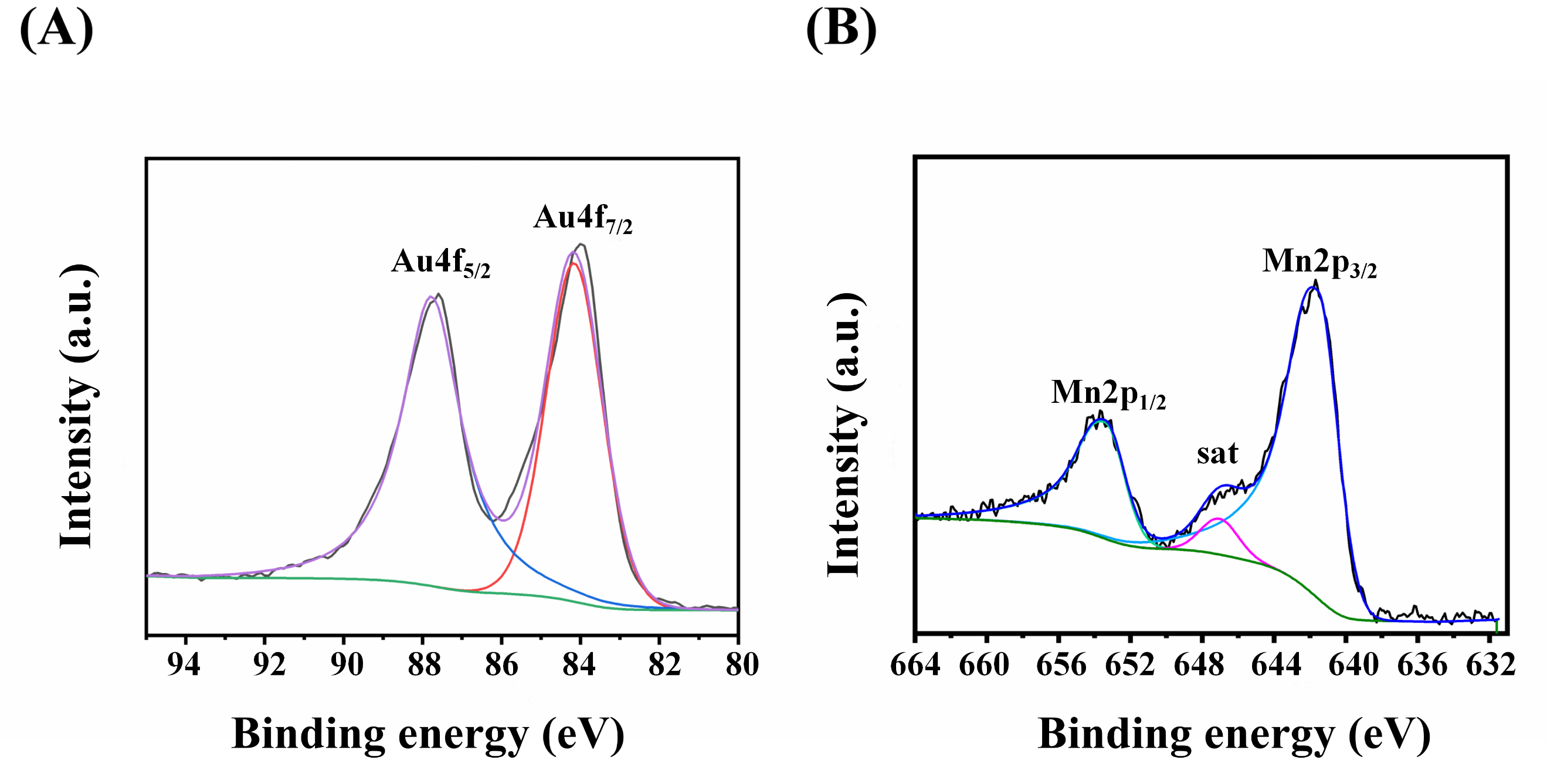


**Figure S2** XPS analysis on the surface chemical composition of MSN-Au@CO. XPS spectra of (A) Au 4 f and (B) Mn 2p.


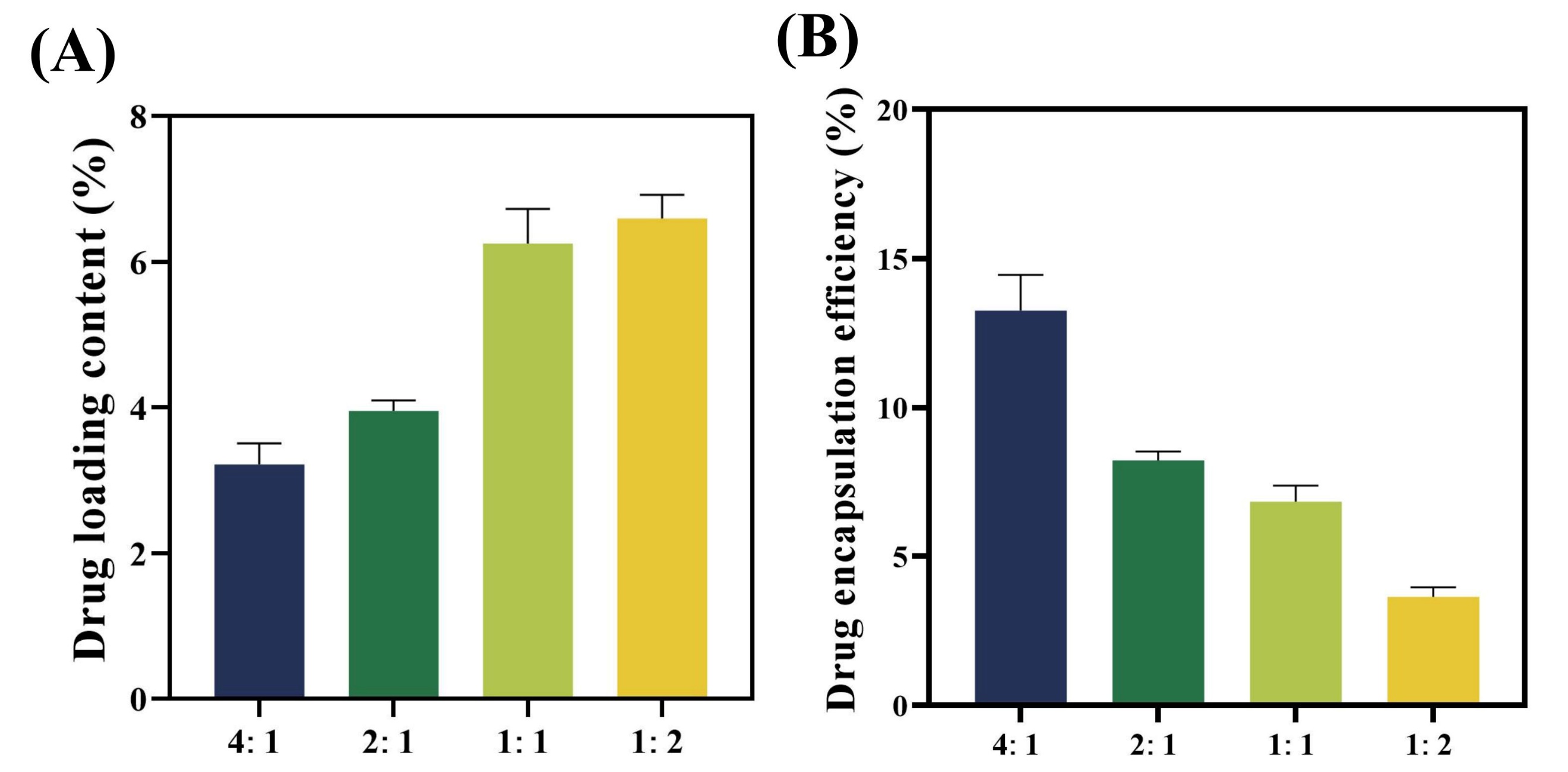


**Figure S3** The (A) drug-loading content, and (B) the drug encapsulation efficiency of MnCO when the mass ratios of MSN-Au and MnCO were 4:1, 2:1, 1:1, and 1:2 respectively.

**
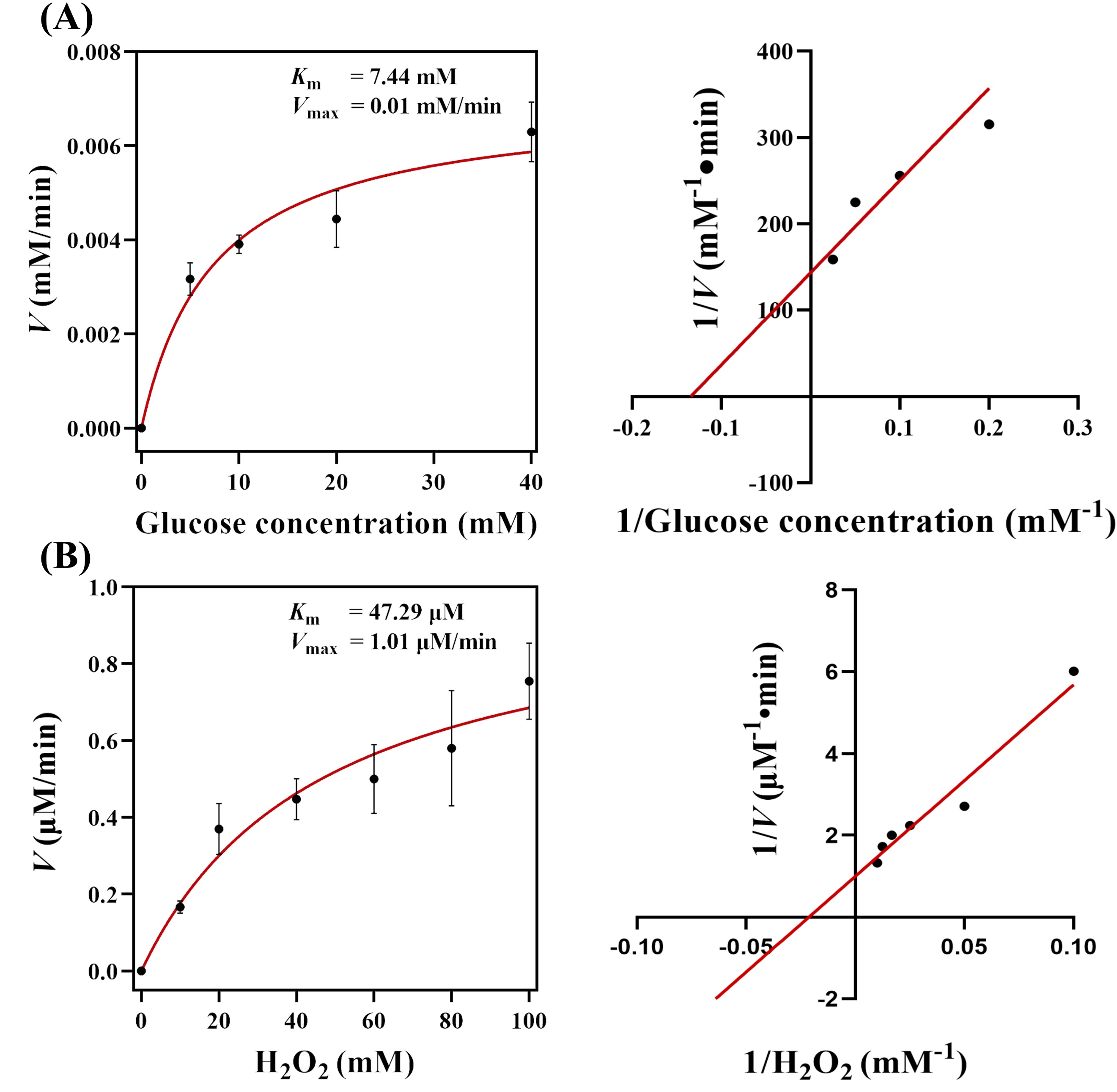
**

**Figure S4** Catalytic kinetics of the (A) GOx-like and (B) POD-like activity of MSN-Au@CO.

**
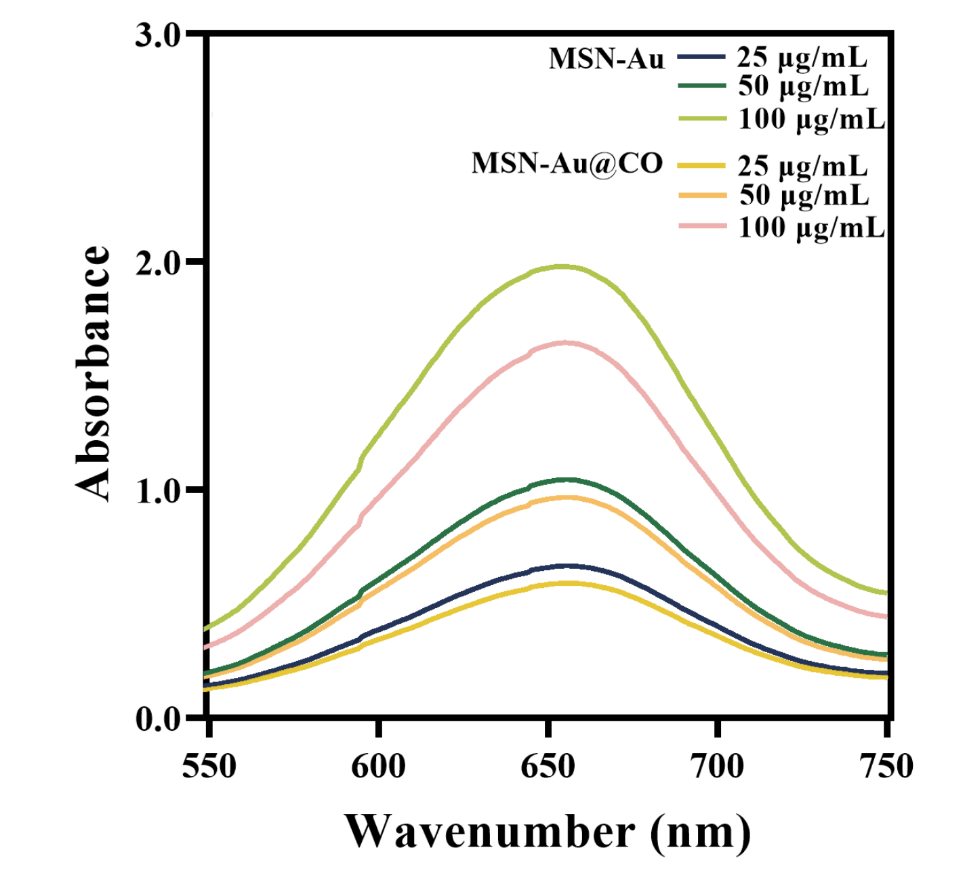
**

**Figure S5** POD-like activity of different concentrations of MSN-Au and MSN-Au@CO.

**
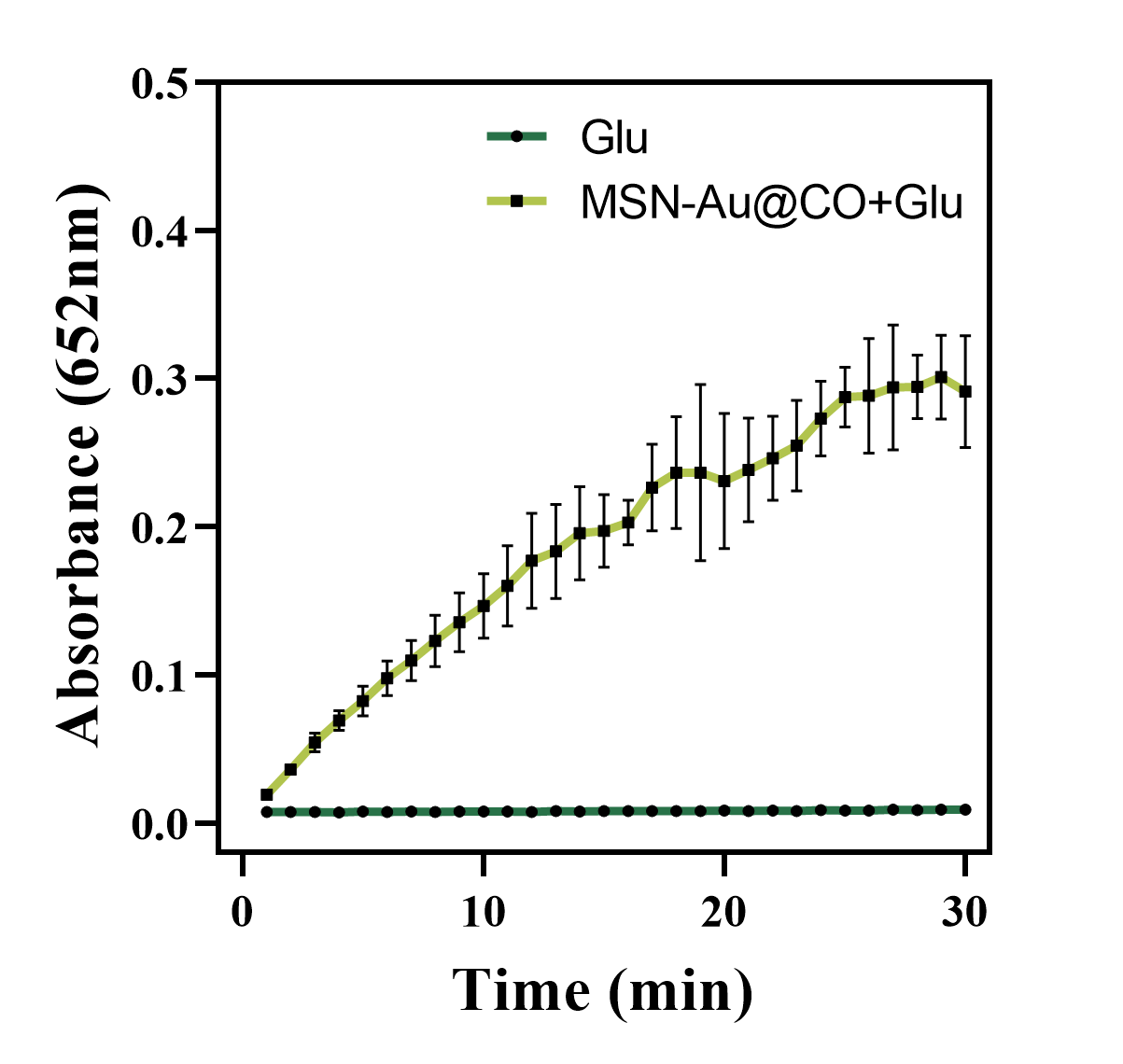
**

**Figure S6** The enzyme cascade reaction activity of MSN-Au@CO. With TMB as the chromogenic substrate, the absorbance of MSN-Au@CO solution in the presence of glucose changed.


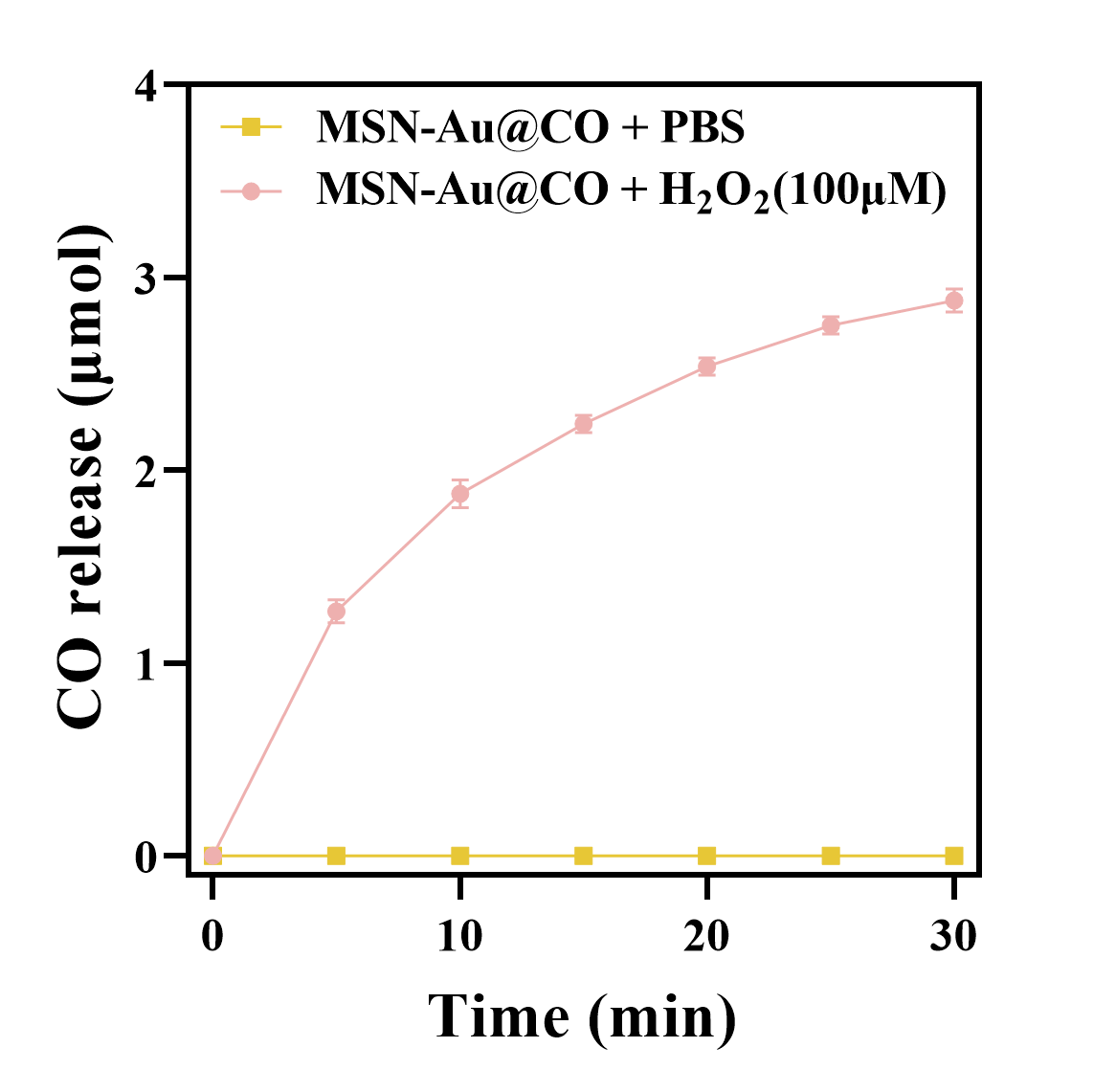


**Figure S7** The ability of MSN-Au@CO to release carbon monoxide with or without hydrogen peroxide (100 μM)


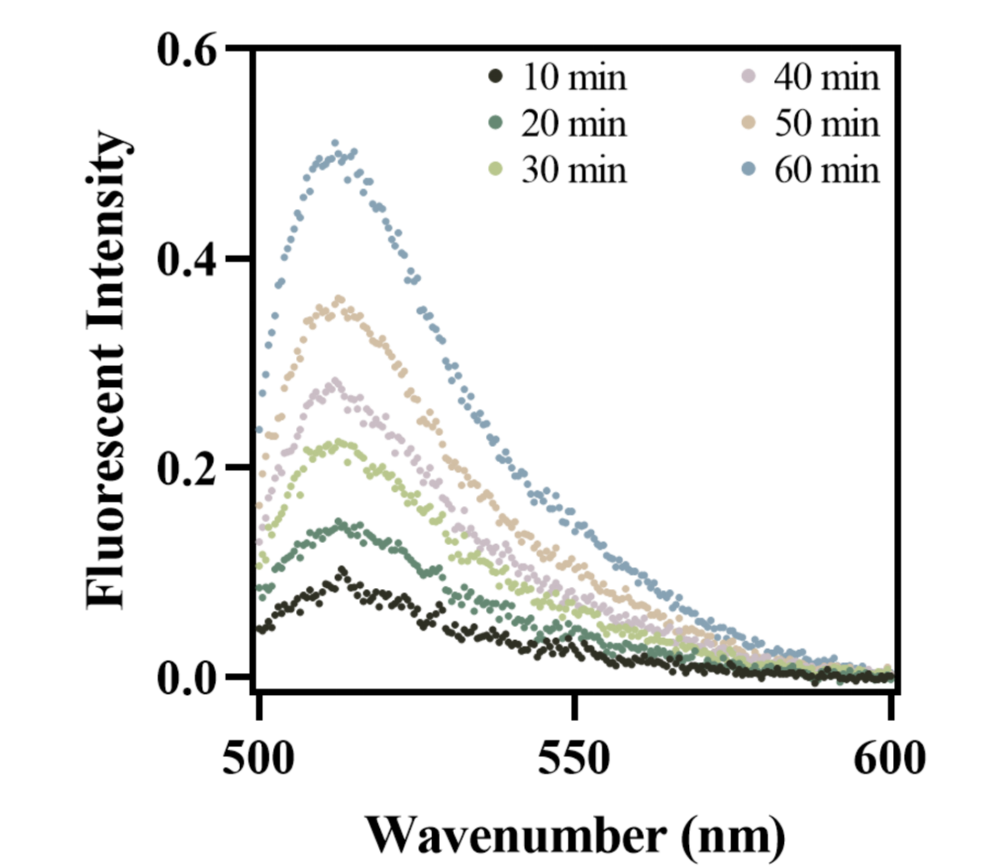


**Figure S8** Fluorescence intensity of carbon monoxide detected by FL-CO-1 fluorescence probe at different time points in MSN-Au@CO (100 μg/mL).

_
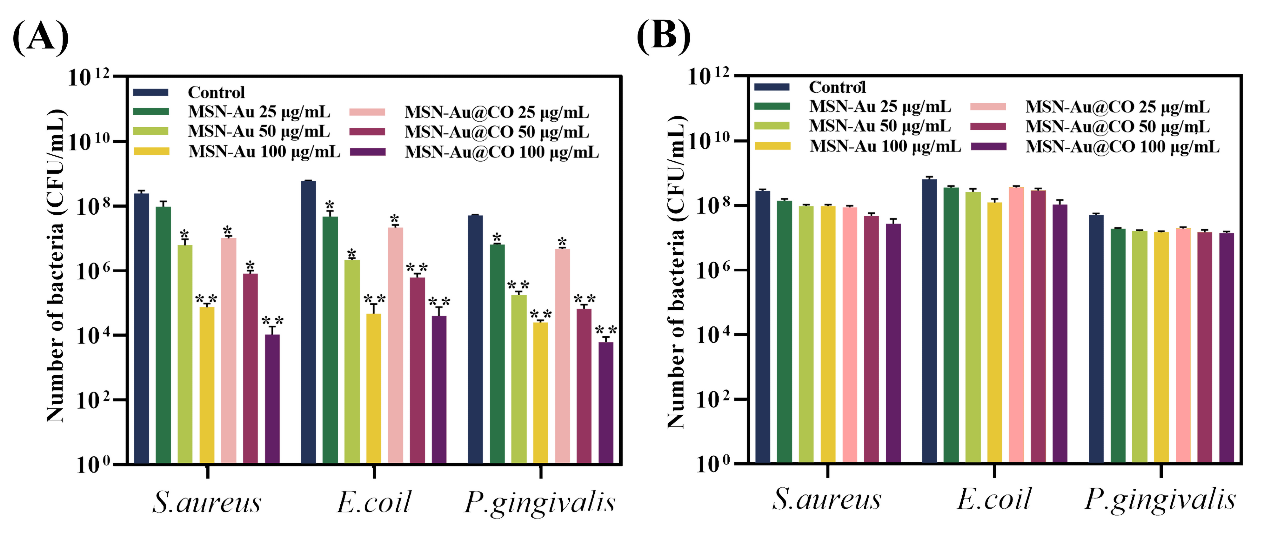
_

**
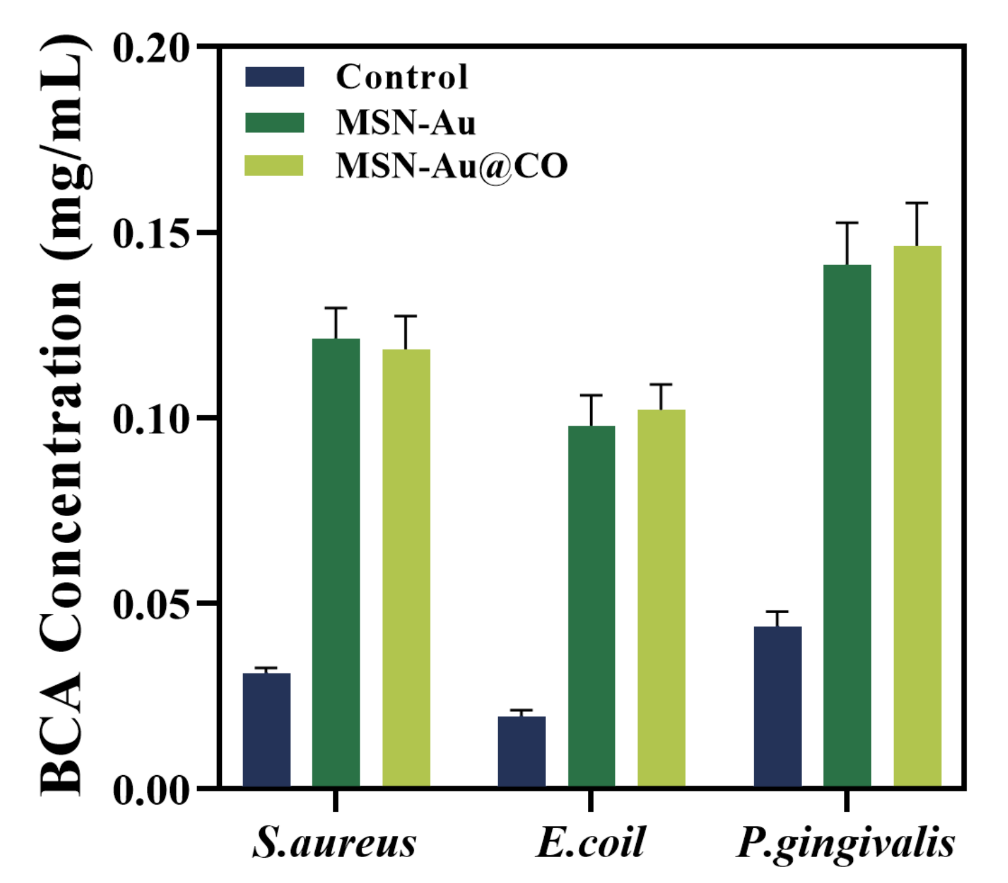
Figure S9** Colony quantification diagram of *S. aureus*, *E. coli*, and *P. gingivalis* after different treatments with (A) and without (B) glucose (**^*^** Compared with the Control group).

**Figure S10** Protein leakage in *S. aureus*, *E. coil,* and *P. gingivalis* after treatment with MSN-Au (50 μg/mL) and MSN-Au@CO (50 μg/mL).


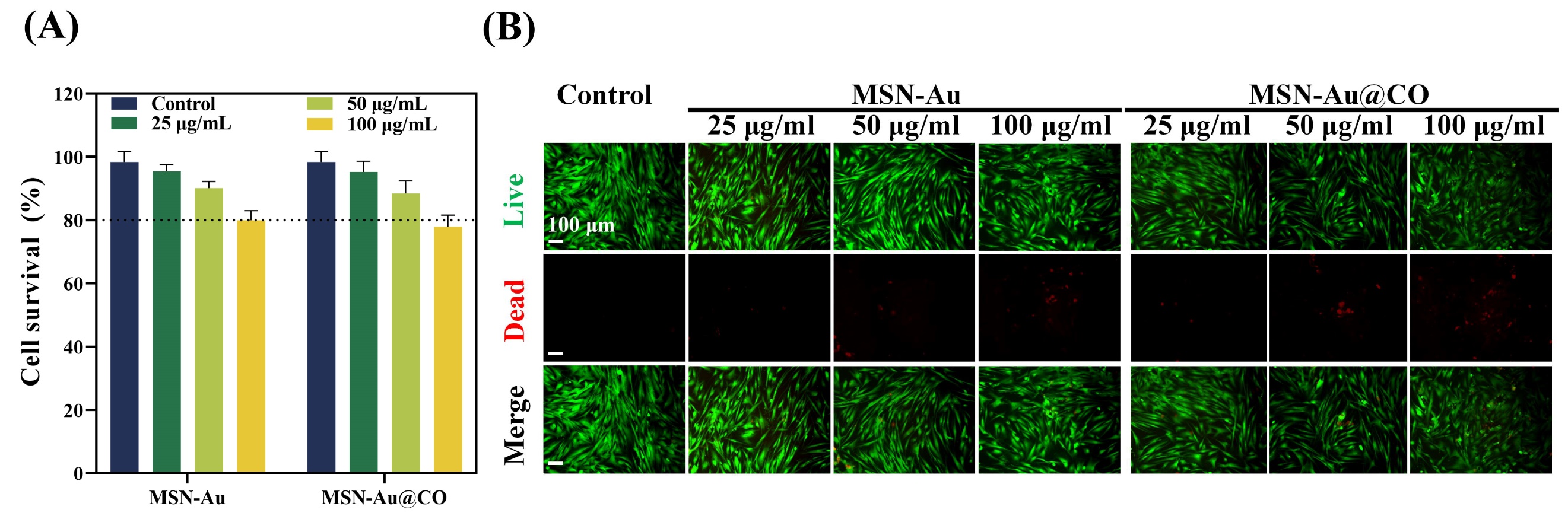


**Figure S11** Biocompatibility of MSN-Au@CO in vitro. (A) Determination of cell viability of human periodontal ligament cells (hPDLCs) by MTT method (48 h). (B) Changes of Live/Dead staining of hPDLCs after various treatments for 24 h.

**
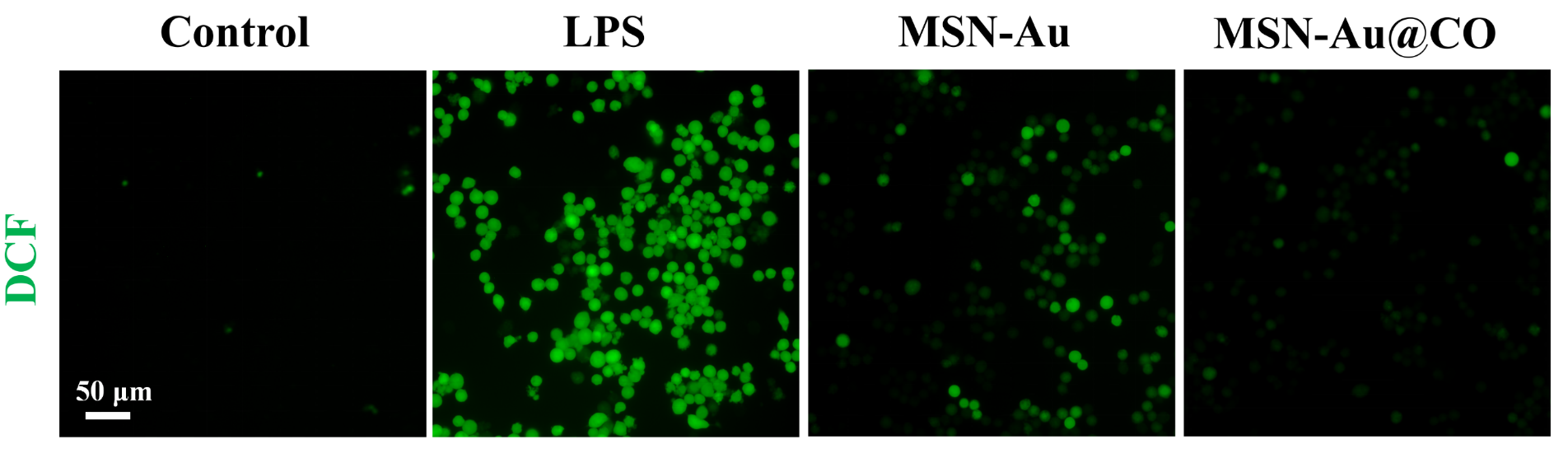
**

**Figure S12** Representative DCFH-DA probe staining images of RAW264.7 cells after different treatments.


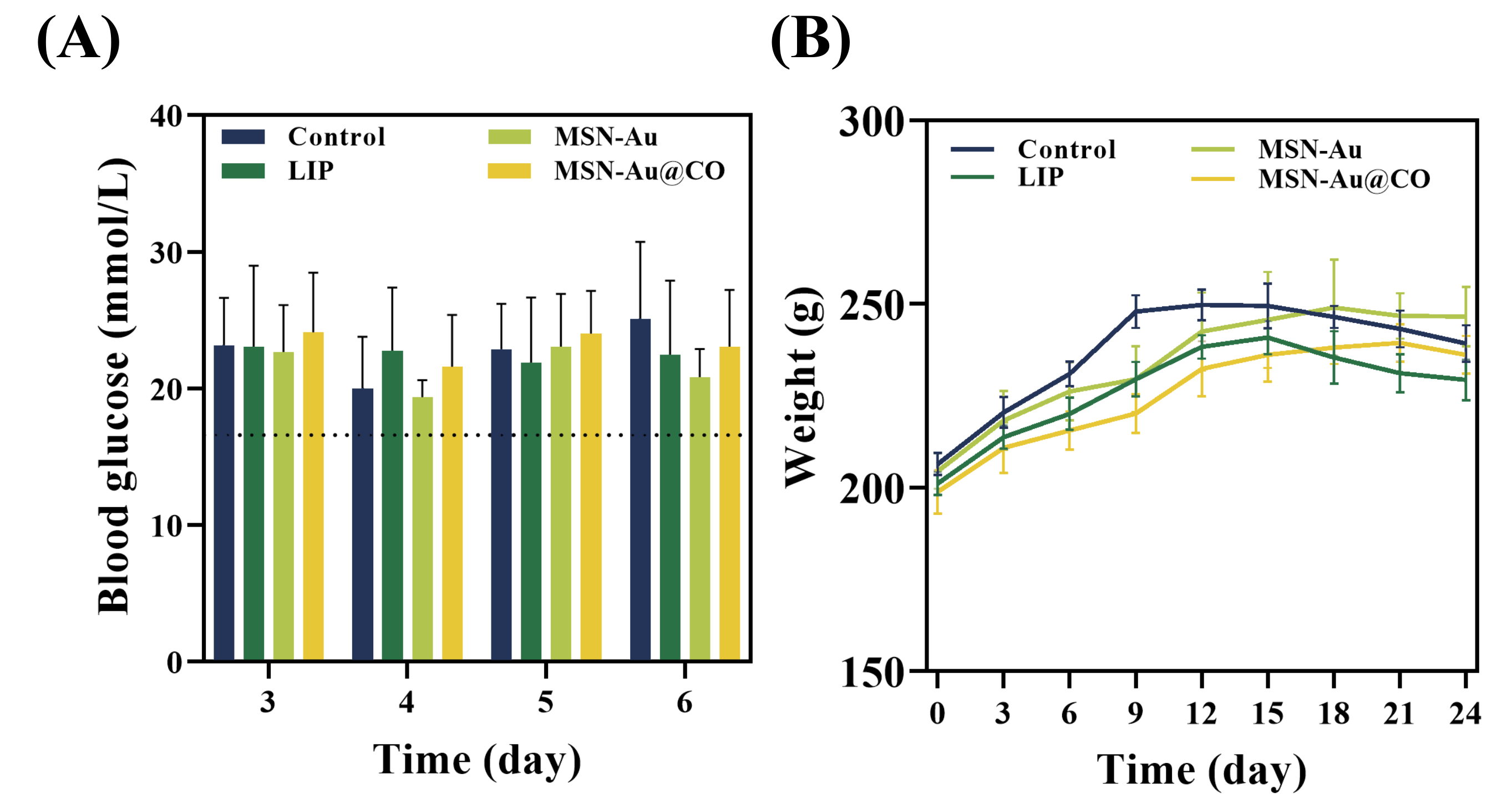


**Figure S13** Changes of (A) blood glucose and (B) body weight in rats after different treatments.


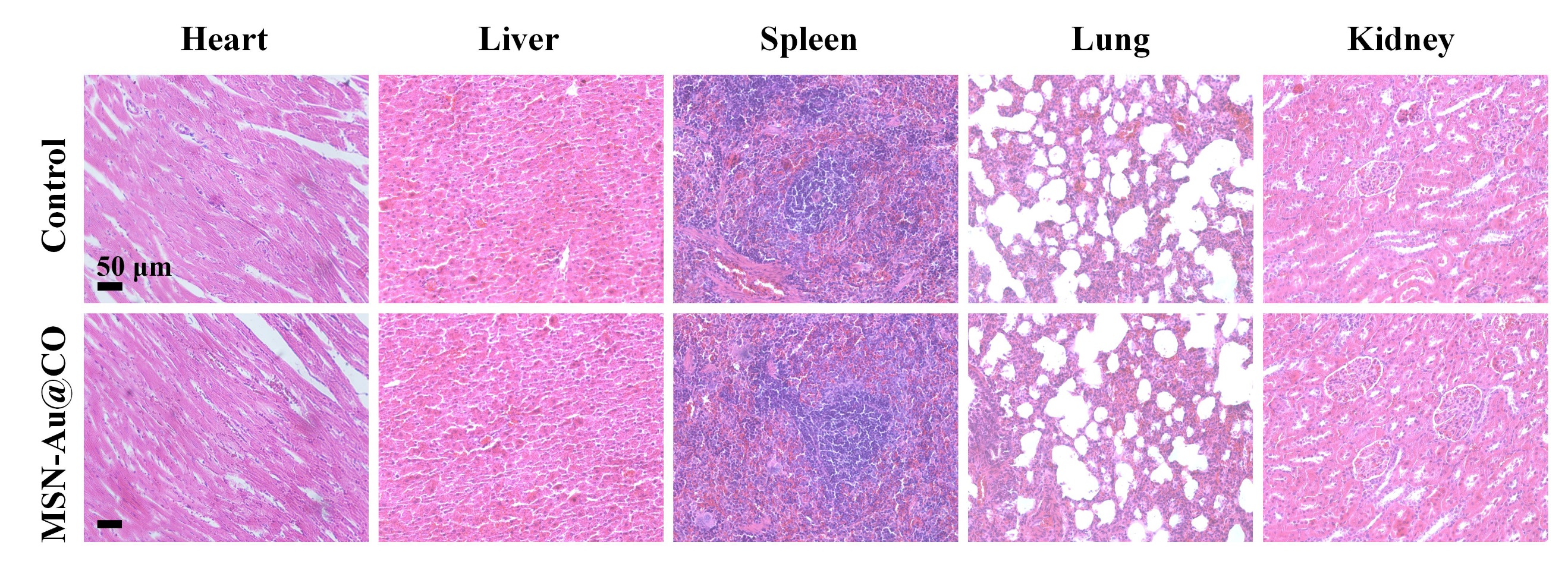


**Figure S14** H&E staining images of major organs collected from rats after MSN-Au@CO treatment.

**Supplemental Tables:**

**Table S1** Lambert Beer's law.

$$\begin{aligned} A=Kbc\#\left( 1 \right) \end{aligned}$$

Note: A refers to the absorbance of the solution, K is the molar absorptivity of Au NPs (2.6×10^^8^ L*mol-1*cm^-1^), b refers to the optical pathand, and c is the molar concentration of Au NPs.

**Table S2** Michaelis-Menten and Lineweaver-Burk equation.

$$\begin{aligned} V=\frac{V_{max}\left[ S \right]}{K_{m}+\left[ S \right]}\#\left( 2 \right) \end{aligned}$$

$$\begin{aligned} \frac{1}{V}=\frac{K_{m}+\left[ S \right]}{V_{max}\left[ S \right]}=\frac{K_{m}}{V_{max}}\frac{1}{\left[ S \right]}+\frac{1}{V_{max}}\#\left( 3 \right) \end{aligned}$$

Note: (2) Michaelis-Menten equation. (3) Lineweaver-Burk transformed the Michaelis-Menten equation. The *K*m is the substrate concentration at which the reaction rate is at half-maximum, [*S*] is the glucose concentration and *V*max is the maximum reaction velocity at the saturating substrate concentrations.

**Table S3** Calculation formula of carbon monoxide concentration.

$$\begin{aligned} N_{CO}=\frac{pV_{g}}{RT}+cV_{l}=p\left( \frac{V_{g}}{RT}+\frac{V_{l}}{k} \right)\#\left( 4 \right) \end{aligned}$$

Note: *p* is the partial pressure of CO, *V*_g_ and *V*_l_ are the volume of the gas phase (850 mL) and liquid phase (2 mL), R is the gas constant (0.08205 L·atm·mol^−1^·K^−1^), T is the temperature,c is the CO concentration in the liquid phase, and k is Henry’s law constant of CO in water (1052.63 L·atm·mol^−1^ at 25 °C).

**Table S4** Calculation formula of hemolysis rate.

Hemolysis ratio (%) = [(ODh-ODn)/(ODp-ODn)] ×100% (5)

Note: ODh, ODn, and ODp indicate the absorbance values of the nanozymes, PBS, and DI water groups, respectively.

**Table S5** The primer sequences used for q-PCR in the study.

| **Gene** | **Gene forward primer sequence (5’-3’)** | **Reverse primer sequences (5’-3’)** |
| --- | --- | --- |
| IL-1β | TGTGCAAGTGTCTGAAGCAGC | TGGAAGCAGCCCTTCATCTT |
| TNF*-*α | CTCATGCACCACCATCAAGG | ACCTGACCACTCTCCCTTTG |
| IL-6 | AGTTGCCTTCTTGGGACTGA | TCCACGATTTCCCAGAGAAC |
| GAPDH | ATCACTGCCACCCAGAAG | TCCACGACGGACACATTG |
